# Remediating Desmoplasia with EGFR‐Targeted Photoactivable Multi‐Inhibitor Liposomes Doubles Overall Survival in Pancreatic Cancer

**DOI:** 10.1002/advs.202104594

**Published:** 2022-06-24

**Authors:** Girgis Obaid, Shazia Bano, Hanna Thomsen, Susan Callaghan, Nimit Shah, Joseph W. R. Swain, Wendong Jin, Xiadong Ding, Colin G. Cameron, Sherri A. McFarland, Juwell Wu, Mark Vangel, Svetla Stoilova‐McPhie, Jie Zhao, Mari Mino‐Kenudson, Charles Lin, Tayyaba Hasan

**Affiliations:** ^1^ Department of Dermatology Massachusetts General Hospital and Harvard Medical School Boston MA 02114 USA; ^2^ Present address: Department of Bioengineering University of Texas at Dallas Richardson TX 75080 USA; ^3^ University of Texas at Arlington Arlington TX 76019 USA; ^4^ Center for Nanoscale Systems Harvard University Cambridge MA 02138 USA; ^5^ Division of Health Sciences and Technology Harvard University and Massachusetts Institute of Technology Cambridge MA 02139 USA

**Keywords:** desmoplasia, molecular targeting, nanomedicine, pancreatic cancer, photodynamic therapy

## Abstract

Desmoplasia is characteristic of pancreatic ductal adenocarcinoma (PDAC), which exhibits 5‐year survival rates of 3%. Desmoplasia presents physical and biochemical barriers that contribute to treatment resistance, yet depleting the stroma alone is unsuccessful and even detrimental to patient outcomes. This study is the first demonstration of targeted photoactivable multi‐inhibitor liposomes (TPMILs) that induce both photodynamic and chemotherapeutic tumor insult, while simultaneously remediating desmoplasia in orthotopic PDAC. TPMILs targeted with cetuximab (anti‐EGFR mAb) contain lipidated benzoporphyrin derivative (BPD‐PC) photosensitizer and irinotecan. The desmoplastic tumors comprise human PDAC cells and patient‐derived cancer‐associated fibroblasts. Upon photoactivation, the TPMILs induce 90% tumor growth inhibition at only 8.1% of the patient equivalent dose of nanoliposomal irinotecan (nal‐IRI). Without EGFR targeting, PMIL photoactivation is ineffective. TPMIL photoactivation is also sixfold more effective at inhibiting tumor growth than a cocktail of Visudyne‐photodynamic therapy (PDT) and nal‐IRI, and also doubles survival and extends progression‐free survival by greater than fivefold. Second harmonic generation imaging reveals that TPMIL photoactivation reduces collagen density by >90% and increases collagen nonalignment by >10^3^‐fold. Collagen nonalignment correlates with a reduction in tumor burden and survival. This single‐construct phototoxic, chemotherapeutic, and desmoplasia‐remediating regimen offers unprecedented opportunities to substantially extend survival in patients with otherwise dismal prognoses.

## Graphical abstract

Desmoplasia in pancreatic ductal adenocarcinoma (PDAC) is one of most significant hurdles in effective management of the disease. Treating desmoplasia alone has proven to be unsuccessful in the clinic, thus necessitating transformative combination approaches. This study is the first to demonstrate that a desmoplasia‐remediating regimen using a single targeted and activable photodynamic‐chemotherapeutic construct doubles overall survival in orthotopic PDAC.

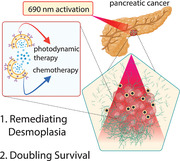

## 1 Introduction

Pancreatic cancer, of which 90% is pancreatic ductal adenocarcinoma (PDAC), is the fourth highest cause of cancer deaths and exhibits dismal 5‐year survival rates (≈9%).^[^ ^1^ ^]^ In metastatic patients, 5‐year survival is down to 3%.^[^ ^1^ ^]^ Desmoplasia is a fibrotic stromal reaction that leads to a significant elevation of extracellular matrix (ECM) deposition in PDAC. Desmoplasia restricts the infiltration of drugs and immune cells, and contributes to chemoresistance, pro‐tumorigenic signaling, tumor progression, and metastasis.^[^ ^2^, ^3^, ^4^ ^]^ Collagen, primarily synthesized by pancreatic cancer‐associated fibroblasts (PCAFs), is the most abundant ECM protein in the PDAC matrisome and contributes to 90% of all ECM signaling during all disease stages.^[^ ^5^ ^]^ Although Hedgehog pathway inhibitors, hyaluronidase, and other stromal therapies can deplete the stroma and potentiate drug delivery in pre‐clinical PDAC models, genetic and pharmacological Hedgehog inhibition in PDAC patients did not improve clinical outcomes.^[^ ^3^, ^6^, ^7^, ^8^, ^9^, ^10^ ^]^

Given the current clinical failures to remediate desmoplasia while controlling tumor progression, and the inability for even the most toxic chemo‐regimen, FOLFIRINOX, to extend median survival beyond 11.1‐months, a critical unmet need for transformative approaches to better manage PDAC remains.^[^ ^11^ ^]^ These include strategies that simultaneously target stromal and cellular PDAC constituents to mitigate desmoplasia‐induced resistance.

Photodynamic therapy (PDT), a light‐based photochemical modality, has demonstrated strong potential in managing desmoplastic PDAC in the pre‐clinical and clinical (Phase I, I/II) setting.^[^ ^12^, ^13^, ^14^, ^15^ ^]^ PDT induces direct tumor tissue photodamage, photodynamic priming (PDP; subtherapeutic doses of PDT), sensitization to combination agents (e.g., chemotherapy, small molecule inhibitors, biologics, and immunotherapeutics), and immunogenic cell death.^[^ ^16^ ^]^ PDP of PDAC has also been shown to mitigate chemo‐induced selection pressures and potentiate chemotherapy outcomes.^[^ ^17^ ^]^ In our recent study using an orthotopic PDAC model comprising both MIA PaCa‐2 PDAC cells and PCAFs, we have shown that PDP using the clinical formulation Visudyne, along with Vitamin D receptor activation in PCAFs, allows for more durable responses to nanoliposomal irinotecan (nal‐IRI) chemotherapy.^[^ ^12^ ^]^ Importantly, PDP along with Vitamin D receptor activation in PCAFs reduced the CXCL12‐CXCR7 paracrine signaling between PCAFs and MIA PaCa‐2 cells, which is critical for tumor progression in desmoplastic tumors.^[^ ^12^ ^]^ With regards to desmoplasia, we have shown that molecular targeted PDT can specifically reduce the tumor collagen fractional area in subcutaneous PDAC models,^[^ ^14^ ^]^ which is largely attributed to photochemical disruption of the ECM.^[^ ^18^ ^]^ In other elegant work, subtherapeutic PDT of subcutaneous prostate tumors also reduced collagen content, thereby potentiating nanoparticle delivery.^[^ ^19^ ^]^ Molecular targeted PDT of stromal cells has also been shown to downregulate their ECM deposition.^[^ ^20^ ^]^ Molecular targeted PDT, termed photoimmunotherapy (Phase III, NCT03769506) when using antibody‐photosensitizer (PS) conjugates,^[^ ^21^, ^22^, ^23^ ^]^ is able to confine phototoxicity to tumor tissue with molecular precision. Molecular targeted PDT increases the tolerance to high‐dose PDT, thereby allowing more complete tumor photodestruction while sparing T cells.^[^ ^14^, ^24^ ^]^

**Figure 1:**
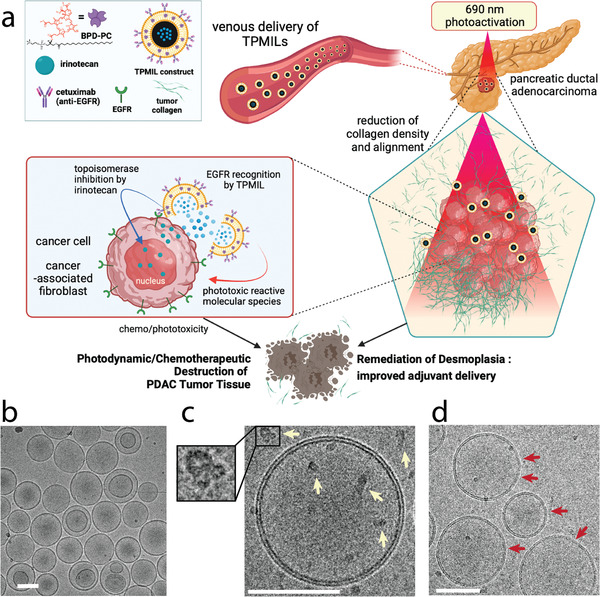
a) Graphical representation of the mechanism of action following photoactivation of TPMIL constructs which provide concomitant EGFR‐specific photodynamic damage of PDAC tumor cells, topoisomerase inhibition by irinotecan, and photodynamic remediation of desmoplasia in orthotopic tumors (figure created with Biorender.com). b) Cryo‐transmission electron microscope (TEM) images of the TPMIL constructs with surface‐bound Cet (off‐white arrows) c) resolved at higher magnifications. The structure of Cet can also be resolved at the TPMIL surface (inset in (c)). d) Cryo‐TEM images of TPMIL constructs following 690 nm photodynamic activation reveals a loss of membrane integrity (red arrows) as a reduction in the contrast of the phospholipid bilayer (scale bars are 100 nm).

This study is the first to remediate desmoplasia in an orthotopic PDAC model containing patient‐derived PCAFs using an EGFR‐molecular targeted PDT‐based combination. We integrate the following salient features into a single therapeutic nanoconstruct and a single treatment protocol in order to provide a transformative desmoplasia‐remediating approach to manage PDAC: 1) an EGFR molecular targeted construct that provides confined tumor‐specific photodamage, 2) co‐encapsulated irinotecan to simultaneously inhibit tumor cell topoisomerase I at substantially de‐escalated chemotherapy doses, and 3) a collagen photo‐modulating approach that decreases collagen density as well as collagen alignment, a negative prognostic indicator in PDAC (**Figure** **1**a).^[^ ^25^ ^]^ The impact of this integrated approach on tumor burden, desmoplasia, and survival are investigated by leveraging the targeted 690 nm‐photoactivable multi‐inhibitor liposomes (TPMILs) developed here in an orthotopic MIA PaCa‐2 + patient‐derived PCAF model of PDAC. Both the MIA PaCa‐2 cells and patient‐derived PCAF cells used in this study overexpress EGFR^[^ ^14^ ^]^ and are thus targeted by a cetuximab (anti‐EGFR mAb)‐functionalized TPMIL construct that co‐encapsulates a lipidated variant of the PS benzoporphyrin derivative (BPD) along with irinotecan chemotherapy. Second harmonic generation (SHG) imaging of orthotopic desmoplastic PDAC tumor cryosections following 690 nm photoactivation of the TPMIL construct is used to investigate the impact on tumor collagen density and collagen alignment in desmoplasia.

## 2 Experimental Section

### 2.1 TPMIL Synthesis

TPMILs containing the conjugate of the PS BPD and 1‐arachidoyl‐2‐hydroxy‐*sn*‐glycero‐3‐phosphocholine (20:0 BPD‐PC), irinotecan, and surface functionalized with a lipid conjugate of cetuximab were prepared using the following process.^[^ ^14^, ^26^ ^]^ 20:0 BPD‐PC was synthesized by esterification of BPD (U.S. Pharmacopeia) and 20:0 lyso PC (Avanti Polar Lipids) using our previously published protocol.^[^ ^26^ ^]^ Cetuximab (Cet 2 mg mL^−1^; Eli Lily) was modified with Alexa Fluor‐488 NHS ester (Promega) and PEG‐4‐Azide NHS ester (ThermoFisher) as have previously described.^[^ ^14^ ^]^

Liposomes were prepared by mixing chloroform solutions of DSPC, cholesterol, DSPE‐mPEG_2000_ (Avanti Polar Lipids), and 20:0 BPD‐PC at a molar ratio of 36.2, 31.3, 0.2, and 0.4, respectively. Chloroform was evaporated using a flow of nitrogen followed by 1 h storage under vacuum. Lipids were then dissolved in 50% w/v ethanol (Sigma Aldrich), heated at 65 °C for 10 min, vortexed then mixed with 10 volumes of preheated 250 mm ammonium sulfate (Sigma Aldrich) followed by 1 min vortexing.^[^ ^27^ ^]^ Hydrated lipids were subjected to 5 freeze‐thaw cycles in liquid nitrogen and 65 °C water at 3‐min and 5‐min intervals, respectively, followed by 15 extrusions through 100 nm polycarbonate membranes at 40 °C. Excess ammonium sulfate was removed using Sepharose CL‐4B equilibrated with HEPES dextrose buffer (5 mm HEPES, 5% dextrose, pH 6.5; Sigma Aldrich). Liposomes were then mixed with irinotecan hydrochloride trihydrate (≈20 mg mL^−1^ in HEPES dextrose buffer; LC Labs) at a ratio of 284.06 g irinotecan/mol phospholipid and heated to 65 °C in the dark for 30 min to facilitate gradient loading of the drug into the liposomes. Liposomes were then cooled on ice for 15 min. The BPD‐PC concentration of irinotecan loaded liposomes was measured using US–vis spectrophotometry (Thermo Scientific Evolution 300).^[^ ^26^ ^]^ DSPE‐mPEG_2000_ and DOPG (Avanti) micelles in HEPES dextrose buffer were post‐inserted into liposomes at a 4.0‐fold and 6.8‐fold molar excess to BPD‐PC equivalent by heating at 65 °C for 1 h, following by cooling on ice for 15 min. The post‐inserted liposomes were buffer exchanged overnight at 4 °C by dialysis in 100 kDa Float‐A‐Lyzer tubes (Spectrum) against HEPES saline buffer (5 mm HEPES, 145 mm NaCl, pH 6.5; Sigma Aldrich).

Cet‐PEG‐Azide was conjugated to DSPE‐PEG_2000_‐DBCO micelles in HEPES saline buffer by an overnight click reaction at room temperature. DSPE‐PEG_2000_‐Cet micelles were then post‐inserted with the liposomes at 100‐to‐1 ratio at 37 °C for 18 h to yield TPMILs. Following cooling on ice for 15 min, the TPMILs were purified from un‐inserted DSPE‐PEG_2000_‐Cet micelles using Sepharose CL‐4B columns. Untargeted PMIL constructs were prepared using the same method without the addition of DSPE‐PEG_2000_‐Cet micelles prior to the final 18 h incubation at 37 °C. The constructs were characterized using dynamic light scattering (Malvern Zetasizer Nano ZS), UV–vis spectrophotometry and fluorimetry (Horiba FluoroMax) from a minimum of three individual preparations. The irinotecan entrapment efficiency was quantified in TPMIL constructs following the final Sepharose CL‐4B size exclusion chromatography purification step using both UV–vis spectrophotometry (Figure S2a, Supporting Information) and by liquid chromatography tandem mass spectrometry (LC‐MS/MS). For quantitation of the irinotecan entrapment efficiency using UV–vis spectrophotometry, the relative concentrations of irinotecan (*ε*_384 nm_ = 21 835 m ^−1^ cm^−1^)^[^ ^17^ ^]^ and BPD‐PC (*ε*_687 nm_ = 34 895 m ^−1^ cm^−1^)^[^ ^26^ ^]^ in the purified TPMIL constructs diluted in DMSO were quantified. The contribution of BPD‐PC absorbance to the irinotecan absorbance maxima at 384 nm was corrected using a correction factor of 0.11 with respect to BPD‐PC absorbance at 689 nm. The following equation was then used to determine the irinotecan entrapment efficiency:

(1) $$\begin{array}{c} \begin{array}{c} \text{Irinotecan}\;\text{loading}\;\text{efficiency}=\frac{\begin{array}{c} \mathrm{Irin}\mathrm{otec}\mathrm{an}\;\mathrm{to}\;\mathrm{BPD}-\mathrm{PC}\;\mathrm{molar}\\ \mathrm{ratio}\;\mathrm{in}\;\mathrm{purf}\mathrm{ied}\;\mathrm{TPMIL} \end{array}}{\begin{array}{c} \mathrm{Irin}\mathrm{otec}\mathrm{an}\;\mathrm{to}\;\mathrm{BPD}-\mathrm{PC}\;\mathrm{molar}\\ \mathrm{ratio}\;\mathrm{in}\;\mathrm{as}-\mathrm{synt}\mathrm{hesi}\mathrm{zed}\;\mathrm{TPMIL} \end{array}}\times \;100 \end{array} \end{array}$$

The irinotecan entrapment efficiency was validated using the Agilent 6430 Triple Quad LC‐MS/MS (586.8 m/z precursor, 124 m/z product ion, 75 fragmentor, 15 collision energy, 7 accelerator voltage, positive ion mode), using napthyl ethylene diamine dihydrochloride (Sigma) as an internal standard.

### 2.2 Singlet Oxygen Quantum Yield Measurement

The singlet oxygen quantum yield of the TPMIL construct (10 µm BPD equivalent in aerated PBS) was determined by measuring the phosphorescence of singlet oxygen (1276 nm) using a PTI Quantamaster spectrometer with a Hamamatsu R5509–42 liquid nitrogen‐cooled photomultiplier tube.^[^ ^28^ ^]^ Ru(bpy)3 in acetonitrile at room temperature was used as a reference.

### 2.3 Phototriggered Irinotecan Release

For all photodynamic activation processes in this study, the duration of light illumination was determined by both the operating power density of the laser (irradiance; mW cm^−2^) and the energy dose depicted as the power density (fluence; J cm^−2^). The relationship between irradiation time, irradiance, and fluence is provided below:

(2) $$\text{Power}\;\text{density}\;\left(W\;{\text{cm}}^{-2}\right)=\frac{\text{Energy}\;\text{density}\;\left(J\;{\text{cm}}^{-2}\right)}{\text{Irradiation}\;\text{time}\;\left(s\right)}$$

and therefore,

(3) $$\text{Irradiation}\;\text{time}\;\left(s\right)=\frac{\text{Energy}\;\text{density}\;\left(J\;{\text{cm}}^{-2}\right)}{\text{Power}\;\text{density}\;\left(W\;{\text{cm}}^{-2}\right)}$$

As‐synthesized TPMIL constructs were diluted in a PBS solution (Corning) containing 10% fetal bovine serum (FBS, Corning) and were equilibrated at 37 °C for 3 h. The constructs in serum‐containing buffer were then activated by 690 nm laser light (high power devices; 40 J cm^−2^, 100 mW cm^−2^) in the absence or presence of the triplet state quencher 100 mm sodium azide (Sigma). Unirradiated TPMIL constructs were kept in the dark. All constructs were then transferred to 30 kDa Float‐A‐Lyzer dialysis tubes (Spectrum) and dialyzed against a PBS solution containing 10% FBS, pre‐equilibrated at 37 °C. Aliquots of TPMIL construct (20 µL) were removed from dialysis at 3, 6, 21, 93, 141, 189, 261, and 309 h after irradiation and stored at −80 °C for characterization. The BPD‐PC concentration in each aliquot was quantified using fluorimetry^[^ ^26^ ^]^ and the irinotecan concentration was measured using the Agilent 6430 Triple Quad LC‐MS/MS as described above.

### 2.4 Confocal Microscopy

MIAPaCa‐2 cells (30 000 cells per well) were seeded in 96 well glass‐bottom plates for 24 h at 37 °C. TPMIL constructs were added to the cells at a concentration of 1 µm BPD‐PC eq. and incubated for 6 h. Before imaging, 10 µL Lysotracker Green DND‐26 (ThermoFisher Scientific), was added to the samples at a working concentration of 50 nm and was incubated for 1 h. Cells were then imaged using an Olympus Confocal Laser Scanning Microscope FV3000 using a 100× oil immersion objective.

### 2.5 TPMIL Stability in Absence and Presence of Cells

MIAPaCa‐2 cells (1500 cells per well) were seeded in 96 well plates. The stability of TPMIL constructs in serum‐containing media was assessed at two different temperatures, 37 (with cells) and 4 °C (without cells). TPMIL construct in DMEM media with 10% FBS (20 µm BPD‐PC eq.) was either stored at 4 °C or added to the cells and incubated for 7 days. The size and polydispersity of the TPMIL constructs was monitored using dynamic light scattering as described above.

### 2.6 In Vitro Cytotoxicity and Phototoxicity

MIAPaCa‐2 cells (3000 cells per well) were seeded in black‐walled transparent based 96 well plates for 24 h at 37 °C prior to the addition of constructs. After 24 h, serum‐containing culture media with diluted constructs were added to each well: TPMIL, PMIL, Visudyne, and nal‐IRI. After 6 h, the constructs were removed from the cells, the cells were washed three times with 200 µL of fresh media. For the cytotoxicity assessment, designated plates were further incubated for 72 h in the dark. For the phototoxicity assessment, designated plates were irradiated with 690 nm light using a fluence of 20 J cm^−2^ then further incubated for 72 h in the dark. The MTT assay (3‐(4,5‐dimethylthiazol‐2‐yl)‐2,5‐diphenyltetrazolium bromide; ThermoFisher Scientific) was then performed where 100 µL of media containing 0.3 mg mL^−1^ of MTT was added into each well and incubated with the cells for 2 h. All the well contents were then removed and 100 µL of DMSO was added to each well. The absorbance of the MTT product, formazan, was then measured at 555 nm using a Tecan plate reader.

### 2.7 Orthotopic MIA PaCa‐2 + PCAF Tumor Implantation

An orthotopic desmoplastic model of PDAC was implanted using MIA PaCa‐2 cells (ATCC Cat# CRM‐CRL‐1420, RRID:CVCL_0428; https://web.expasy.org/cellosaurus/CVCL_0428) and patient‐derived PCAF cells (kind gift from Dr. Simeone^[^ ^29^ ^]^) in Athymic male Swiss nu/nu mice (4–6 weeks old, 20 g, COX7 Animal Facility). All animal procedures were performed in accordance with approved Institutional Animal Care and Use Committee (IACUC) Protocol Number 2006N000084. MIA PaCa‐2 cells and PCAF cells were cultured in DMEM media with glutamine (Corning), 10% FBS and 1× penicillin/streptomycin (Gibco). Mycoplasma‐free cells (MycoAlert Detection Kit (Lonza)) were trypsinized and suspensions (50 µL) containing 1 × 10^6^ cells of each of MIA PaCa‐2 cells and PCAF cells were prepared in 1:1 culture media‐to‐Matrigel (BD Biosciences). Athymic male Swiss nu/nu mice were randomly assigned to treatment groups prior to tumor implantation. Following therapy, mice were assigned blinded code numbers where the investigators could not match subjects to treatment groups. The selection of animal numbers was based on power calculations that achieved a 0.05 two‐sided significance level (*p* < 0.05) at 80% statistical power using a two‐tailed *t*‐test to detect changes in tumor volumes (Cohen's d) and estimates of population standard deviations based on previously published data.^[^ ^12^ ^]^ Once differences in tumor volumes reached statistical significance, no further animals were used.

Inclusion criteria for mice were an age of 4–6 weeks, a body weight that did not exceed ±10% of 20 g, healthy activity levels and eating habits, no signs of aggression or infection, and tumors that developed at day 9 after implantation. Mice that did not meet the inclusion criteria were excluded from the study and were humanely euthanized. Animal subject attrition was only in the case of animals that did not develop tumors following implantation (implantation failure ≈10%) and those that developed unrelated infections (≈2%).

Randomized mice were anesthetized with ketamine‐xylazine and underwent a laparotomy procedure to externalize the pancreas. 50 µL suspensions of MIA PaCa‐2 + PCAF cells were implanted in the head of the pancreas, after which the pancreas was returned and the animal sutured with 4‐0 polyglycolic acid absorbable sutures (AD Surgical). Pre‐operative and post‐operative buprenorphine was administered as per approved IACUC protocols.

### 2.8 TPMIL Micro‐Distribution and Full Body Biodistribution

**Figure 2:**
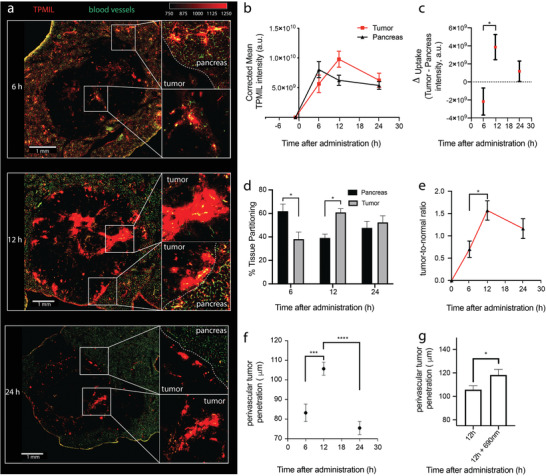
TPMIL constructs exhibit maximal tumor tissue selectivity and penetration 12 h following intravenous administration. a) TPMIL (red, 0.5 mg kg^−1^) microdistribution in MIA PaCa‐2 + PCAF orthotopic tumors and surrounding pancreatic tissue with respect to vasculature (green). b) TPMIL mean intensity is highest in the pancreas at 6 h and highest in the tumor at 12 h after intravenous administration. c) The greatest different between tumor uptake and pancreatic tissue uptake is at 12 h after intravenous administration. d) TPMIL partitioning in the tumor tissue, e) TPMIL tumor‐to‐normal ratio, and f) TPMIL perivascular penetration are all greatest at 12 h after intravenous administration. g) Photoactivation of TPMIL 12 h following administration increases perivascular penetration by ≈11% (values are mean ± S.E.M.; statistical significance was calculated using one‐way ANOVA with a Tukey post‐test; *n* = 6; *p ≤ 0.05, ***p ≤ 0.005, ****p ≤ 0.001).

Mice were administered with TPMIL construct 9 days following tumor implantation, and were sacrificed at 6, 12, or 24 h post administration. Mice were intravenously injected with 100 µL Dyelite488‐labeled tomato lectin (1 mg mL^−1^; Vector Laboratories) 5 min prior to sacrifice to label blood vessels. Tumor‐bearing pancreases were harvested, bisected along the center of the tumor, embedded in CRYO‐OCT Compound (Fisher), and cryosectioned onto glass slides at a thickness of 10 µm. Entire tumor cross sections and surrounding pancreatic tissue were then imaged using an Olympus FV1000 confocal microscope at 10× 5 µm optical planes. BPD‐PC in the TPMIL constructs and Dyelite488‐tomato lectin was visualized with 405 and 488 nm lasers, respectively. ImageJ (ImageJ, RRID:SCR_003070; https://imagej.net/) was used to quantify TPMIL tissue signals. Perivascular diffusion was quantified using the ImageJ radial profile tool. Raw images were used for all analysis. For visual clarity only, the red channel was set to 750–1250, and the green channel was set to 2978.56–5652.08 (6 h) and 1800–4100 (12 and 24 h) in **Figure** **2**.

Upon determining that the optimal TPMIL tumor microdistribution was at 12 h following administration, mice were administered with TPMIL construct (0.5 mg kg^−1^ BPD eq.) as described above, and were sacrificed at 12 h post administration. The tumor, heart, kidneys, lung, liver, pancreas, muscle, and spleen were harvested and imaged using hyperspectral imaging on the Maestro Imaging System (Perkin Elmer). BPD‐PC signals from the TPMIL constructs were corrected by spectrally unmixing from healthy tissue autofluorescence to quantify relative tissue uptake.

### 2.9 In Vivo Therapy

9 days following implantation, randomized mice were intravenously administered with the following agents corresponding to the treatment groups in the results section, as described in **Table** **1** below. nal‐IRI was obtained from Ipsen and Visudyne was obtained from Bausch + Lomb.

**Table 1:** Description of dosimetry of PDT agents and chemotherapy used for the in vivo tumor therapy studies

Treatment Arm	Injected dose of active agent [mg kg^−1^]^a)^	Light fluence [J cm^−2^]	PDT dose product [mg kg^−1^ BPD eq.] × [J cm^−2^]
TPMIL	0.25 BPD eq.; 9.60 IRI eq.	0	0.0
	0.25 BPD eq.; 9.60 IRI eq.	50	12.5
	0.50 BPD eq.; 19.20 IRI eq.	100	50.0
	0.75 BPD eq.; 28.80 IRI eq.	150	112.5
PMIL	0.25 BPD eq.; 9.60 IRI eq.	50	12.5
Visudyne	0.25	50	12.5
	0.75	150	112.5
nal‐IRI	5.00	0	n.a.
	20.00	0	n.a.
LOW IRI TPMIL	0.25 BPD eq.; 1.5 IRI eq.	50	12.5

^a)^ The dose equivalents of both BPD‐PC and IRI are specified in the TPMIL and PMIL constructs that co‐encapsulate both agents.

TPMIL, PMIL, and Visudyne was photoactivated with 690 nm light (Laser, High Power Devices; 100 mW cm^−2^) at 12 h, 12 h, or 90 min following administration, respectively, using the same laparotomy described above. Tumor volume was longitudinally monitored using the Vevo LAZR ultrasound and photoacoustic tomography system (VisualSonics) until they became moribund or if the tumors reached 20 mm in any single dimension, at which point the mice were euthanized humanely. Progression‐free survival was calculated by interpolating survival days at which tumors were greater than or equal to 100 mm^3^ from a Malthusian exponential growth fit of mean tumor volumes. Outlier tests were performed using the GraphPad Prism online tool and statistical significance was calculated using GraphPad Prism 8.4.2 (GraphPad Prism, RRID:SCR_002798; http://www.graphpad.com/).

### 2.10 Second Harmonic Generation Imaging

SHG imaging was performed on 20 µm cryosections of treated or untreated tumors 72 h after initiation of therapy. SHG imaging was performed on the Olympus FV1000 multiphoton microscope using a tunable Mai Tai laser at 880 nm, a 40× water objective and a 400–460 nm SHG emission filter set. Random sampling of 10 ROIs from each bisected tumor section was performed (*n* = 4–5 mice; 40–50 ROIs per arm) at a minimum of 200 µm away from the boundary between the tumor and pancreas.

Images were analyzed using ImageJ with a FIJI processing package (Fiji, RRID:SCR_002285; http://fiji.sc) and MATLAB (MATLAB, RRID:SCR_001622; http://www.mathworks.com/products/matlab/). Orientation information was analyzed using the plugin OrientationJ (OrientationJ, RRID:SCR_014796, http://bigwww.epfl.ch/demo/orientation/) in “analysis” mode. Tiff files were imported and subjected to a bandpass filter (large structures filtered to 1060 pixels and small structures filtered to 6 pixels) and background subtraction (rolling ball radius; 25). A binary image was created using thresholding (min; 18, max; 255). The original, non‐binary, images were entered into OrientationJ, which evaluates the structure tensor at every pixel and local orientation properties found with the equations found in the OrientationJ Manual (PDF). Orientation information was collected using the “RGB” setting with the red channel set to “Orientation,” and the resultant image was multiplied by the binary image to isolate orientation information at only the pixels correlating to collagen fiber signals. Histogram data were collected in counts from 0 to 255, corresponding to counts in the range of 0°–180^○^. Variance was calculated using MATLAB and statistical analysis was measured in GraphPad.

Collagen fiber density by area was calculated by the percent of the total image covered by pixels corresponding to collagen fiber, calculated with binary image histogram data via MATLAB. Collagen fiber intensity was calculated in ImageJ as integrated density. Briefly, a binary image was created by setting a threshold (mentioned above) to isolate collagen fiber signal. The binary image was converted to a mask which was reapplied to the original image, containing intensity values, such that collagen fiber intensity was isolated only from pixels corresponding to fibers. Integrated density and mean intensity were collected using ImageJ measurements. Outlier tests were performed using the GraphPad Prism online tool and statistical significance was calculated using GraphPad as described above.

### 2.11 Statistical Analysis

1. Pre‐processing of data was only performed for the circular angular variance in the SHG imaging SHG data, were the collagen fiber circular variance was normalized to (collagen fiber area)^2^. Evaluation of outliers was performed using the GraphPad Prism outlier test using a significance level of 0.05.
2. Details of all data presentation are defined within each figure legend, and are largely mean ± S.E.M.
3. Sample sizes (*n*) for each statistical analyses are defined for each dataset in the respective figure legends.
4. Animal numbers were selected based on power calculations using a two‐tailed *t*‐test for a *p* < 0.05 significance level at 80% statistical power. Statistical significance all data in this study were analyzed for statistical significance using a one‐way analysis of variance (ANOVA) test with a Tukey multiple comparisons post‐hoc test. All data comparisons in the study were individually assigned a *p* value as defined in the respective figure legends. No alpha adjustments were made.
5. GraphPad Prism v9 was used for all statistical analyses in this study
6. Figure legends: Please make sure that all relevant figure legends contain the information on sample size (*n*), probability (*p*) value, the specific statistical test for each experiment, data presentation, and the meaning of the significance symbol.

## 3 Results

TPMIL constructs were prepared by actively loading irinotecan using an ammonium sulfate gradient. Irinotecan is a topoisomerase I inhibitor that induces tumor cell cycle arrest and apoptosis. The TPMIL membrane contained a lipidated derivative of the clinical PS BPD (20:0 BPD‐PC).^[^ ^26^ ^]^ Post‐insertion of micellular DSPE‐PEG_2000_‐Cetuximab into the TPMILs conferred molecular specificity toward EGFR overexpressed on MIA PaCa‐2 PDAC and PCAF membranes. We initially compared the therapeutic efficacy of TPMIL constructs prepared using BPD‐PC derivatives with varying lipid chain lengths (16:0 BPD‐PC and 20:0 BPD‐PC) in MIA PaCa‐2 + PCAF 3D nodules and found that 20:0 BPD‐PC was more efficacious (Figure S1a–c, Supporting Information). Furthermore, nodule phototoxicity of TPMILs post‐inserted with the anionic lipid DOPG rendered them more than twice as efficacious (Figure S1a–c, Supporting Information). As such, 20:0 BPD‐PC and DOPG post‐insertion were used for the remainder of the study. Singlet oxygen is one of the primary reactive molecular species generated by BPD‐PC present within the TPMIL constructs.^[^ ^16^, ^26^ ^]^ Using the phosphorescence of singlet oxygen at 1276 nm, the singlet oxygen quantum yield of the TPMIL constructs in aerated PBS was found to be 0.05 ± 0.02. DOPG is an unsaturated phospholipid that reacts with singlet oxygen and is transformed to 9‐ and 10‐hydroperoxides.^[^ ^30^, ^31^, ^32^ ^]^ Not only do these hydroperoxides destabilize the TPMIL membrane to trigger the release of irinotecan, they are redox‐active compounds that can lead to peroxidative injury to the cell.^[^ ^32^ ^]^ Lipid peroxidation may also trigger lipid peroxidation‐mediated stress signaling and lipid radical chain reactions, both which can result in apoptosis. As such, it is most likely that the oxidation products of DOPG are responsible for the increase in therapeutic efficacy observed when activated by 690 nm light (Figure S1c, Supporting Information).

Untargeted PMIL constructs were synthesized using the same method without the final step of micellular DSPE‐PEG_2000_‐Cetuximab post‐insertion. Cryo‐transmission electron microscopy (TEM) of the TPMIL constructs is shown in Figure 1b–d. At higher magnifications, the Cet targeting ligands can be observed and structurally resolved, as can the loss in membrane integrity following 690 nm irradiation (Figure 1b,c). The TPMILs exhibited up to 34.3‐fold binding specificity to MIA PaCa‐2 cells (Figure S1d, Supporting Information). It is well established that MIA PaCa‐2 PDAC cells express 0.6–1.7 × 10^5^ EGFR molecules per cell.^[^ ^14^, ^33^, ^34^, ^35^ ^]^ We have also previously identified that the PCAF cells used in this study express 3.5 × 10^4^ EGFR per cell.^[^ ^14^ ^]^ In previous work, we have shown that Cetuximab targeting of liposomes containing BPD‐PC correlates linearly with EGFR expression levels in cells with varying degrees of EGFR expression.^[^ ^14^ ^]^ These include A341 (2–4 × 10^6^ EGFR per cell), OVCAR‐5 (4 × 10^5^ EGFR per cell), T47D (7 × 10^3^ EGFR per cell), and CHO‐WT (EGFR null) cells. In this study, binding of the TPMIL constructs to MIA PaCa‐2 cells was attenuated significantly when the cells were pre‐treated with free Cetuximab, further confirming the binding specificity of the constructs toward cancer cell EGFR. The physical and chemical properties of the construct are summarized in Table S1, Supporting Information (4–6 preparations). The TPMIL constructs were loaded with high irinotecan payloads (1:41.4 ± 0.04 BPD‐to‐irinotecan mass ratios) with an entrapment efficiency of 92.4%. The TPMIL constructs remain stable for up to 7 days incubated at 4 and 37 °C in biological media containing 10% FBS in the absence and presence of MIA PaCa‐2 cells, with no evidence of increased hydrodynamic diameters or polydispersity indices (Figure S2a,b, Supporting Information). The characteristic absorption band of irinotecan and the characteristic Soret and Q‐bands of BPD‐PC are all visible in the single TPMIL construct following purification (Figure S2c, Supporting Information). When incubated with MIA PaCa‐2 cells, the TPMIL constructs internalize into cells through receptor mediated endocytosis, as confirmed by their sequestration in endolysosomal compartments (Figure S2d, Supporting Information). This is not unexpected, as we have previously shown that liposomes containing phospholipid conjugates of PSs accumulate in endolysosomal compartments both with and without receptor targeting.^[^ ^14^, ^33^, ^37^, ^38^ ^]^

Immediately following 690 nm photodynamic activation with 40 J cm^−2^, the TPMIL constructs destabilized, which progressed for 5 days following irradiation, suggesting irreversible bilayer disruption as a mechanism for phototriggered irinotecan release (Figure S3a,b, Supporting Information). Phototriggered irinotecan release from the TPMIL constructs in 10% FBS solutions at 37 °C was quantified using LC‐MS/MS upon 40 J cm^−2^ photodynamic activation with 690 nm light (Figure S3c–e, Supporting Information). We have previously shown that the phototriggered release of the water soluble drug surrogate calcein disodium salt from liposomes containing the lipid conjugates of IRDye700DX or BPD‐PC (as in the TPMIL construct presented here) depends on the fluence (and thus irradiation time) applied.^[^ ^14^, ^16^ ^]^ The ability for unsaturated phospholipids, such as DOPG that is post‐inserted into the TPMIL constructs, to assist in phototriggered drug release within liposomal membranes is well established.^[^ ^31^ ^]^ DOPG, and other phospholipids with unsaturated double bonds are particularly susceptible to oxidation. As mentioned above, singlet oxygen reacts with unsaturated phospholipids, such as DOPG, to transform them to 9‐ and 10‐hydroperoxides.^[^ ^30^, ^31^, ^32^ ^]^ Hydroperoxides of unsaturated phospholipids, such as DOPG, are more polar than the native lipid.^[^ ^30^ ^]^ As such, oxidation of DOPG into a hydroperoxide results in a destabilized phospholipid bilayer that triggers the release of irinotecan, in addition to increasing its therapeutic efficacy as described above. Following 690 nm activation, the quantity of remaining irinotecan in the TPMIL constructs was periodically measured until 310 h of incubation in 10% FBS solutions at 37 °C. It was found that 690 nm activation of the TPMIL constructs increased the rate of irinotecan release by 21.2 times (Figure S3c, Supporting Information) and increased accumulative irinotecan release by 58.5% (Figure S3e, Supporting Information). Importantly, phototriggered release was prevented by the presence of the singlet oxygen quencher sodium azide, further corroborating the fact that the mechanism of phototriggered release is an entirely photochemical process. These findings are also consistent with our prior work where we have shown that phototriggered release of the small molecule inhibitor cabozantinib and the water soluble fluorophore calcein disodium salt using BPD and BPD‐PC embedded liposomes is an entirely photochemical process.^[^ ^14^, ^36^, ^37^ ^]^

The in vitro (photo)therapeutic efficacy of the constructs in MIA PaCa‐2 cells is summarized in Table S2, Supporting Information. Results show that without light, the TPMIL construct (IC_50_ = 237.07 ± 75.64 nm) provides a 4.9‐fold greater degree of cytotoxicity, as compared to the untargeted PMIL construct (IC_50_ = 1167.67 ± 256.54 nm). With 690 nm photoactivation, the TPMIL construct (IC_50_ = 3.53 ± 0.92 nm) provides a marked 21‐fold greater degree of phototoxicity, as compared to the untargeted PMIL construct (IC_50_ = 74.29 ± 25.80 nm). It was also found that nal‐IRI was significantly less cytotoxic than the PMIL construct in the absence of light. This is most likely due to the enhanced affinity of irinotecan to the liposome carrier in the Ipsen nal‐IRI formulation, which is prepared using a sucrose octasulfate gradient approach.^[^ ^27^ ^]^

The tumor tissue accumulation, microdistribution, and selectivity and peri‐vascular penetration of TPMILs was imaged using confocal microscopy on tumor cryosections harvested at 6, 12, and 24 h following administration (Figure 2a). Pancreatic tissue uptake of TPMIL constructs was highest at 6 h, while tumor uptake was highest at 12 h following administration (Figure 2b). The greatest difference in TPMIL uptake, tissue partitioning, and tumor‐to‐normal ratios between tumor and healthy pancreatic tissue was also found to be at 12 h following administration (Figure 2c–e). The tumor‐to‐normal selectivity of the TPMIL constructs was found to be ≈1.5‐fold at 12 h (Figure 2c). Considering that clinical manifestations of PDAC are characteristically hypovascular,^[^ ^39^ ^]^ and that our orthotopic MIA PaCa‐2 + PCAF tumor model also exhibits 6.99 (±0.33) times lower vascularity than the surrounding pancreatic tissue, this degree of selectivity is in fact commendable. On a per‐vessel basis, the effective tumor selectivity of the TPMIL therefore becomes 10.49‐fold tumor‐to‐normal ratio. Irrespective of tumor selectivity, photodynamic tumor destruction using TPMILs is a threshold phenomenon, whereby the therapeutic reactive molecular species deposited in tumor cells are not limited to local tumor delivery of the construct. Light dosimetry, a critical parameter of PDT based therapies allows therapeutic reactive molecular species to be generated in a catalytic manner. Thus, treatment efficacy becomes primarily dictated by the specificity of the TPMIL construct for tumor tissue EGFR. Furthermore, this study brings out additional powerful attributes of PDT based therapies, whereby stromal disruption as well as vascular modulation assists in drug delivery.

The 12 h timepoint was also found to have the highest TPMIL perivascular penetration, reaching a maximum of ≈105 mm, with penetration being further promoted by 11% with photodynamic activation in a single administration cycle. As such, an administration‐photoactivation interval of 12 h was selected for all TPMIL constructs for the remainder of this study (Figure 2f,g). Full‐body biodistribution of the TPMIL constructs was performed at 12 h following administration using hyperspectral imaging. Results revealed that TPMIL content was greatest in the skin shortly followed by the tumor, and tumor‐to‐normal tissue selectivity was as high as 7.5‐fold with respect to heart tissue (Figure S4a,b, Supporting Information).

In this study, activation of the TPMIL construct containing the BPD‐PC PS molecules was performed using 690 nm laser light, as per the clinical trials on PDAC patients pre‐administered with liposomal BPD.^[^ ^13^, ^40^ ^]^ 690 nm light lies in the far red to near‐infrared region of the electromagnetic spectrum, a region oftentimes referred to as the tissue optical window due to its ideal tissue penetrating properties.^[^ ^16^ ^]^ These two completed clinical studies revealed that 690 nm light can penetrate and damage up to 15 mm of pancreatic tumor tissue in patients. In these clinical studies, light is delivered to pancreatic tumors using endoscopy‐guided fiber optics^[^ ^40^ ^]^ or delivered percutaneously to the tumor through a fiber optic guided by computed tomography.^[^ ^13^ ^]^ The zone of photodamage of PDAC tumor tissue can be further extended in patients when using up to three fibers simultaneously.^[^ ^13^ ^]^ In the clinic, the power density of the 690 nm lasers used in PDAC patients is 150 mW cm^−1^. In this study, a power density of 100 mW cm^−2^ was selected. It must be noted that these laser power densities are non‐thermal and non‐ablative. Unlike thermal lasers, PDT use power densities that are several orders of magnitude lower as the process is entirely photochemical. In the clinic, 200 J cm^−2^ is the highest light dose administered to PDAC patients and only induces tissue phototoxicity in the presence of a PS.^[^ ^13^ ^]^ In this study, a light fluence as high as 150 J cm^−2^ of 690 nm laser light does not impact healthy tissue where TPMIL accumulation is minimal (Figure S4c, Supporting Information). The already‐established clinical irradiation protocols for 690 nm irradiation in PDAC patients significantly increases the translational impact of the TPMIL constructs presented here. Furthermore, clinical results are now pointing toward the fact that the priming effect of PDT extends further beyond the zones of necrosis, and when incorporating the immunological consequences of PDT and PDP, disease management can be further extended even to distant regions.^[^ ^41^, ^42^ ^]^

**Figure 3:**
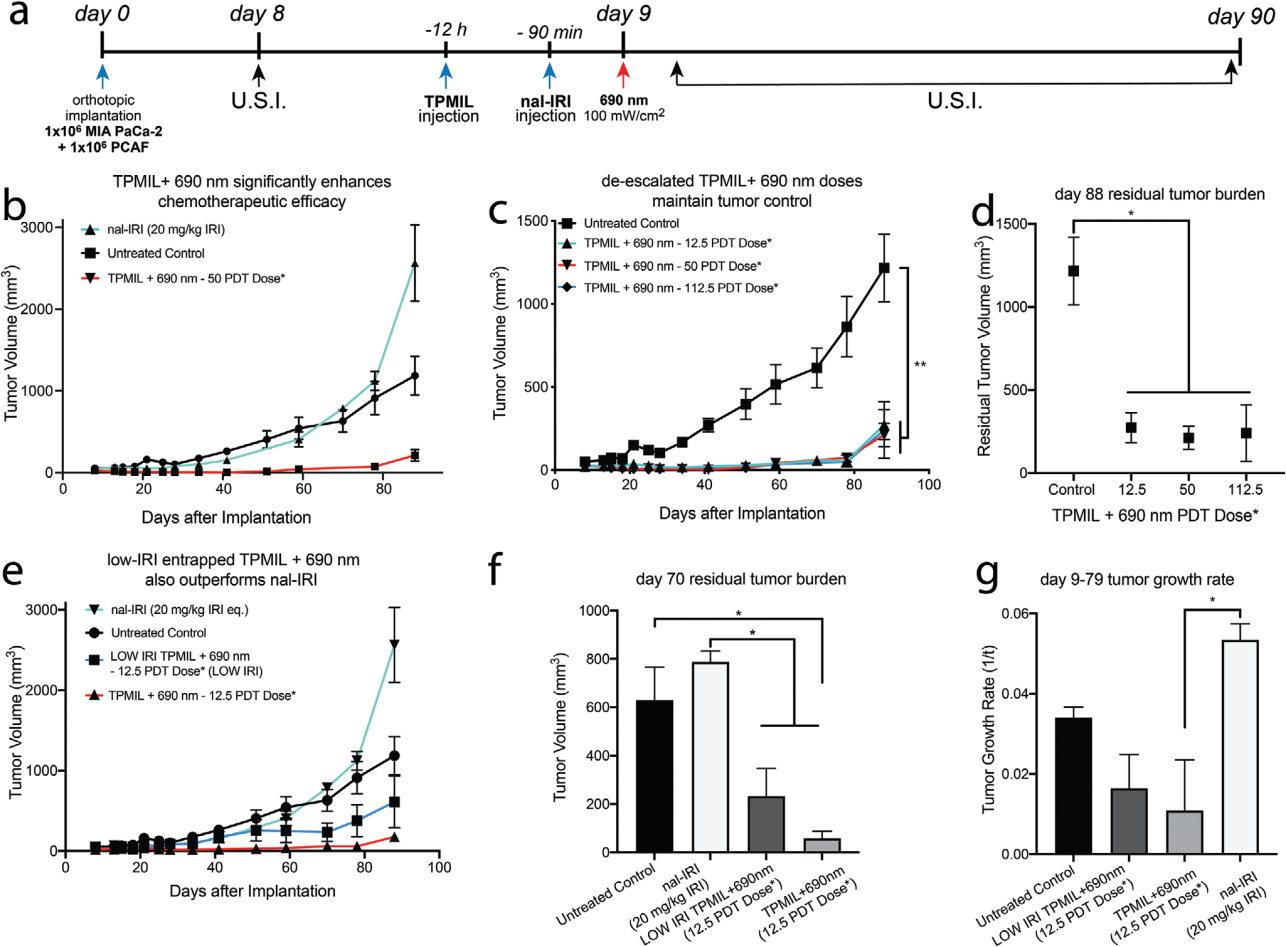
Photodynamic activation of TPMIL controls tumor progression at substantially de‐escalated irinotecan chemotherapy dosages. a) Sequence of MIA PaCa‐2 + PCAF orthotopic tumor implantation, treatment with TPMIL or nal‐IRI and longitudinal tumor volume monitoring using ultrasound imaging (U.S.I.). b) MIA PaCa‐2 + PCAF tumor growth is inhibited by ≈12‐fold following photoactivation of TPMIL constructs containing 19.2 mg kg^−1^ irinotecan, as compared to 20 mg kg^−1^ irinotecan eq. of nal‐IRI alone. c,d) De‐escalated TPMIL PDT dose products remain equally as effective at tumor growth inhibition. e,f) The LOW IRI TPMIL construct encapsulating low doses of irinotecan (1.5 mg kg^−1^ when BPD eq. is 0.25 mg kg^−1^) still remains significantly more effective at controlling tumor growth than nal‐IRI (20 mg kg^−1^). g) Photoactivated TPMIL (12.5 PDT dose product) significantly reduces tumor growth rate as compared to nal‐IRI treatment alone (*PDT dose refers to the dose product of mg BPD eq. kg^−1^ × J cm^−2^; values are mean ± S.E.M.; statistical significance was calculated using one‐way ANOVA with a Tukey post‐test; *n* = 4–7; *p ≤ 0.05, **p ≤ 0.005).

Nal‐IRI is the first approved liposomal nanomedicine for patients with metastatic PDAC and is given in combination with 5‐FU and leucovorin every 2 weeks at a human dose of 70 mg m^−2^ (23.3 mg kg^−1^ mouse eq.) for 9 weeks. This amasses to a total median mouse equivalent dose of 118.2 mg kg^−1^ (FDA Label ID: 3836766). The dosimetry of all treatment arms describing the PDT dose products (mg kg^−1^ BDP eq. × J cm^−2^) and chemotherapy used for the in vivo tumor therapy studies is detailed in Table 1. **Figure** **3**a outlines the scheduling of the PDAC treatment regimens performed in this study. It was found that a single 20 mg kg^−1^ dose of nal‐IRI did not control orthotopic MIA PaCa‐2 and PCAF tumor growth and in fact increased the tumor growth rate with respect to an untreated control (Figure 3b,g). This is consistent with our previous findings that nal‐IRI alone can promote the aggressiveness of MIA PaCa‐2 PDAC tumors by enriching CD44 cancer stem‐like cell subpopulations, an effect that is reversed by PDP.^[^ ^17^ ^]^ However, 19.2 mg kg^−1^ irinotecan encapsulated within TPMIL constructs delivering with a PDT dose product of 50 mg kg^−1^ × J cm^−2^ inhibited tumor growth by 93% with respect to the same dose of nal‐IRI alone (Figure 3a). It was found that TPMIL constructs delivering PDT dose products of 12.5, 50, or 112.5 mg kg^−1^ × J cm^−2^ were all capable of controlling tumor growth (Figure 3c,d). Thus even the lowest TPMIL PDT dose product of 12.5 mg kg^−1^ × J cm^−2^ provided 88.3% tumor growth inhibition with respect to untreated tumors. The light fluence used for the lowest TPMIL PDT dose product of 12.5 mg kg^−1^ × J cm^−2^ (50 J cm^−2^) is even lower than the fluence used for sub‐phototoxic PDP in our previous study, whereby 0.25 mg kg^−1^ BPD eq. and a fluence of 75 J cm^−2^ at an irradiance of 100 mW cm^−2^ exerted no inhibitory effect on tumor growth.^[^ ^12^ ^]^ Photoactivation of the LOW IRI TPMIL construct (12.5 PDT dose product) entrapping ≈13‐fold lower irinotecan concentrations than the nal‐IRI regimen still induced a significant 58.3% tumor growth inhibition with respect to nal‐IRI alone (Figure 3e–g). The regular TPMIL regimen (9.6 mg kg^−1^ IRI eq.; 12.5 PDT dose product), which efficiently inhibited tumor growth (volume and rate), was selected for the remainder of the study. The significance of this effective TPMIL dose regimen is that it comprises only 8.1% of the median total patient dose equivalent of irinotecan.

**Figure 4:**
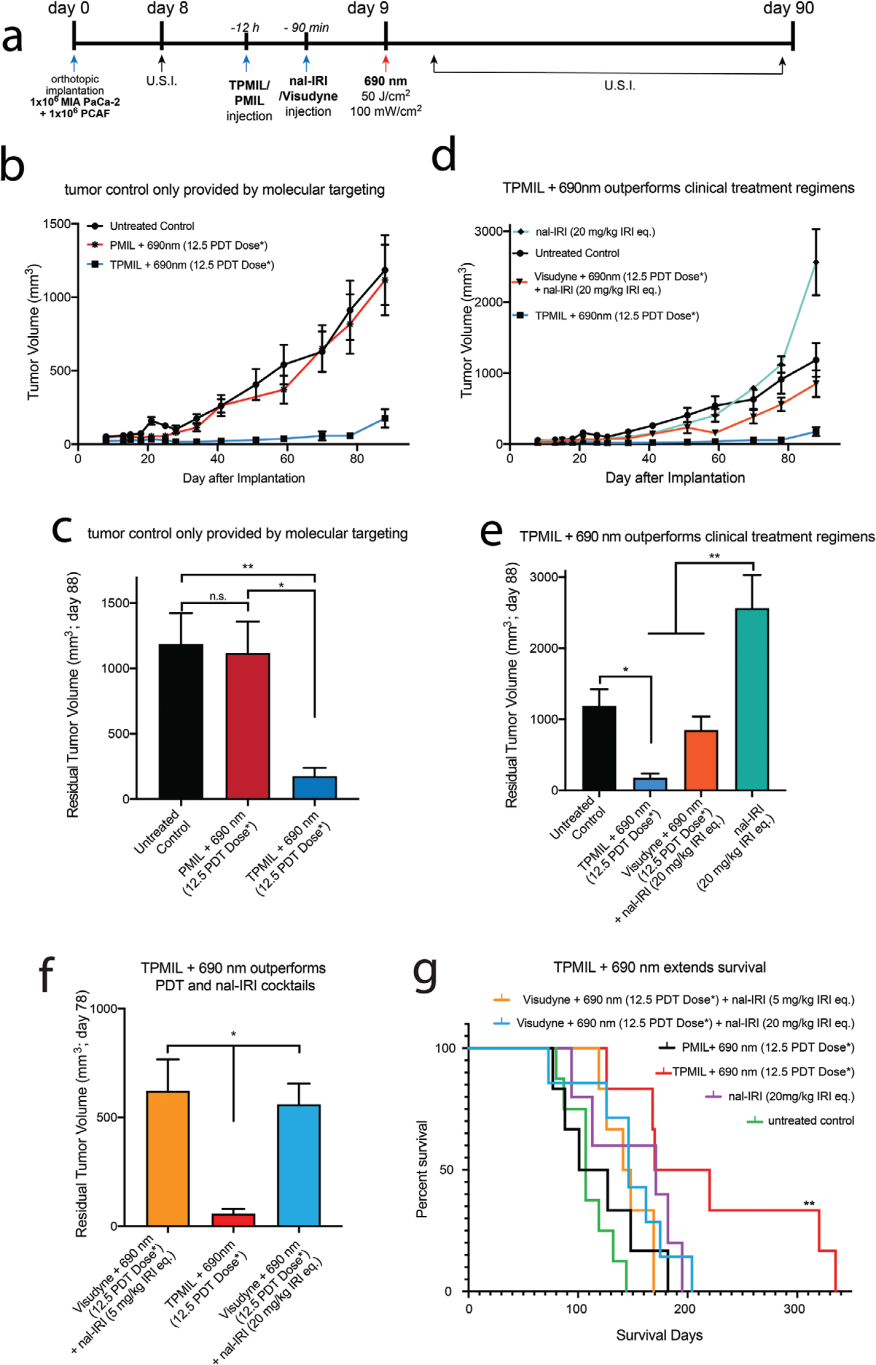
Tumor growth inhibition is only achieved with EGFR targeting of TPMILs, which outperforms clinical regimens of PDT and nal‐IRI, and double survival. a) Sequence of MIA PaCa‐2 + PCAF orthotopic tumor implantation, treatment with TPMIL, PMIL, nal‐IRI, Visudyne, and longitudinal tumor volume monitoring using ultrasound imaging (U.S.I.). b,c) In the absence of molecular targeting of EGFR, the 690 nm‐activated PMIL construct had no inhibitory effect on tumor growth, whereas the targeted TPMIL counterpart induced an approximately tenfold inhibition in tumor growth with photoactivation. d,e) 690 nm‐activated TPMIL constructs were significantly more effective at inhibiting tumor growth than a clinically‐relevant dose of nal‐IRI or a combination of 690 nm‐activated Visudyne and nal‐IRI. f) 690 nm‐activated TPMIL constructs were more effective at controlling tumor growth than a combination therapy of 690 nm‐activated Visudyne PDT with 5 mg kg^−1^ nal‐IRI, and more effective than same combination therapy of 690 nm‐activated Visudyne PDT with a clinically‐relevant nal‐IRI dose of 20 mg kg^−1^ IRI. g) 690 nm‐activated TPMIL constructs extended mice survival more than all other treatment groups (*PDT dose refers to the dose product of mg BPD eq. kg^−1^ × J cm^−2^; values are mean ± S.E.M.; statistical significance was calculated using one‐way ANOVA with a Tukey post‐test (b–f); statistical significance of survival (g) was calculated using a Log‐rank (Mantel–Cox) test; *n* = 4–8; *p ≤ 0.05, **p ≤ 0.005).

Prior elegant work has shown that photo‐triggered release of irinotecan from porphyrin‐doped liposomes within the vasculature of subcutaneous PDAC tumors leads to enhanced tumor deposition of chemotherapy and improved outcomes.^[^ ^43^ ^]^ In our study, the motivating feature of the TPMIL approach lies fundamentally in the molecular specific interactions with tumor receptor EGFR, and tumor cell co‐delivery of both BPD‐PC and irinotecan. As such, we compared the tumor growth inhibition of TPMIL + 690 nm (12.5 PDT dose product) with an untargeted equivalent PMIL construct + 690 nm (12.5 PDT dose product). To our surprise, photoactivation of PMIL resulted in no tumor growth inhibition at the PDT and irinotecan doses used in this regimen. TPMIL + 690 nm photoactivation however led to almost complete tumor growth inhibition, underscoring the criticality of molecular targeting in our approach (**Figure** **4**b,c). It was also found that combining nal‐IRI (20 mg kg^−1^) with Visudyne + 690 nm (12.5 PDT dose product) was still 5.6‐fold less effective at inhibiting tumor growth than TPMIL + 690 nm (12.5 PDT dose product) with only 50% of the irinotecan (Figure 4d,e). The same trend was also consistent when a de‐escalated nal‐IRI dose (5 mg kg^−1^) was combined with Visudyne + 690 nm (12.5 PDT dose product), which was 7.3‐fold less effective at inhibiting tumor growth than TPMIL + 690 nm (12.5 PDT dose product; Figure 4f). These findings underscore the significance of using a single‐nanoconstruct, receptor targeted TPMIL to deliver both chemotherapy and PDT specifically to tumor cells, where cocktails of the same therapies administered using separate nanoconstructs fail to deliver the same degree of tumor control. More importantly, cocktails of the same therapies administered using separate nanoconstructs but with more than threefold higher chemotherapy doses, are still not as effective as the single‐nanoconstruct TPMIL + 690 nm (12.5 PDT dose product). These results are consistent with our prior report of using a single construct containing the PS BPD and the small molecular weight inhibitor cabozantinib, which outperforms cocktails of the two agents together.^[^ ^36^ ^]^ The results of this study emphasize the criticality of delivering combination therapies through a single nanoconstruct, which following molecular targeted PDT, is more efficacious in a tumor environment that has become more conducive to tumor control. The results also emphasize the dependence of molecular targeting on the unprecedented efficacy of the single‐agent photoactivable TPMIL nanoconstruct.

The results underscore the importance of the synergy between co‐encapsulated irinotecan and BPD‐PC, Cetuximab targeting and 690 nm light irradiation. First, the nal‐IRI alone treatment does not suppress tumor growth (Figure 3b). Considering that the TPMIL construct contains less than half of the irinotecan dose than the nal‐IRI alone arm, the tumor growth inhibition observed in the TPMIL + 690 nm arm (12.5 PDT dose product) cannot be attributed to chemotherapy action alone. Second, our prior studies have shown that the same dose of BPD in Visudyne form used in this study is incapable of inhibiting PDAC tumor growth, even when activated with 1.5‐fold higher light dose than that used in this study.^[^ ^12^, ^17^ ^]^ As such, the tumor growth inhibition observed in the TPMIL + 690 nm arm (12.5 PDT dose product) cannot be attributed to PDT alone either. Furthermore, the cocktail regimen of Visuyne‐PDT (12.5 PDT dose product) and nal‐IRI (20 mg kg^−1^) at more than twice the dose of irinotecan in the TPMIL construct was still less than 3 times as effective at inhibiting tumor growth than the TPMIL + 690 nm arm (12.5 PDT dose product; Figure 4d). It is thus imperative that both modalities are integrated into a single targeted TPMIL construct in order to provide the greatest therapeutic benefit. Third, the administered mouse dose of Cetuximab equivalent within the TPMIL construct (0.25 mg kg^−1^ BPD equivalent) is 1.7 mg kg^−1^ Cetuximab equivalent. This equates to 5.1 mg m^−2^ human equivalent dose of Cetuximab, which is 440‐fold lower than the typical human‐equivalent accumulative Cetuximab dose given to patients (Cetuximab FDA Label Reference ID: 4422941). The Cetuximab equivalent dose of TPMIL is therefore only a microdose of the typical therapeutic dose. As such, in the absence of light, the TPMIL construct does not inhibit tumor growth; 690 nm light activation is therefore critical (Figure S5, Supporting Information). With regards to the 690 nm light, it is important to clarify that the laser intensities used for PDT are 2–3 orders of magnitude lower than those used for photothermal therapy.^[^ ^16^ ^]^ A distinguishing attribute of PDT is that it is a cold photochemical modality that only results in tumor tissue damage when a tumor‐localized PS is activated by light. This is further demonstrated by the new in vivo data presented in Figure S5, Supporting Information, which shows that tumor treatment with 50 J cm^−2^ of 690 nm light in the absence of the TPMIL construct does not prevent tumor progression. Therefore, it is only when the TPMIL construct that integrates PDT and irinotecan chemotherapy is activated by 690 nm light that the marked tumor growth inhibition is observed.

In addition to providing the most potent tumor control, mouse survival was the longest in the TPMIL + 690 nm group (12.5 PDT dose product; Figure 4g, Table S3, Supporting Information), which is double that of the untreated control group. For the TPMIL + 690 nm group (12.5 PDT dose product), progression free survival was 78.5 days (209.3% of Visudyne + 690 nm (12.5 PDT dose product) + nal‐IRI (20 mg kg^−1^) cocktail group), median survival was 195.0 days (133.6% of Visudyne + 690 nm (12.5 PDT dose product) + nal‐IRI (20 mg kg^−1^) cocktail group), and overall survival was 335.0 days (164.2% of Visudyne + 690 nm (12.5 PDT dose product) + nal‐IRI (20 mg kg^−1^) cocktail group). It is important to note that with nal‐IRI alone treatment, the mice survive longer than untreated mice even though the tumor volumes with the nal‐IRI alone treatment are larger and, as per our previous data show, the CD44 cancer stem‐like cell subpopulations are enriched.^[^ ^17^ ^]^ The reason is that primary tumor volume is not the only factor affecting survival; many other factors can contribute to the cause of death, including metastatic disease burden. Our previous work has shown that even though nal‐IRI alone treatment does enrich CD44 cancer stem‐like cell subpopulations, it does significantly reduce overall metastatic burden with respect to untreated mice.^[^ ^17^ ^]^ This effect is not surprising, as nal‐IRI is currently approved for PDAC patients and has been shown to significantly extend patient survival.

Acute toxicity was also assessed in mice treated with a high PDT dose; 150 J cm^−2^ fluence and a TPMIL dose of 0.75 mg kg^−1^ BPD‐PC equivalent (112.5 PDT dose product). In these mice, tissue damage was confined to the tumor, while nearby healthy pancreas and spleen tissue was unaffected, and no acute toxicity was observed at 72 h following treatment (Figure S4c,d, Supporting Information). However, when using the same high PDT dose of 150 J cm^−2^ fluence and 0.75 mg kg^−1^ BPD equivalent using Visudyne (112.5 PDT dose product), photodamage was observed in regions of healthy pancreas and spleen tissue, and 50% of the mice suffered acute toxicity 72 h following treatment (Figure S4c,d, Supporting Information). These findings strongly suggest that tumor tissue specificity of the TPMIL constructs increases the treatment tolerability, thereby safely enabling escalated PDT doses as high as 112.5 PDT dose product when attempting complete tumor photodestruction. The results also suggest that 150 J cm^−2^ of 690 nm light does not affect healthy tissue, such as the normal pancreas, where accumulation of the TPMIL construct is minimal (Figure 2a). All tumor volumes were monitored by ultrasound imaging using the Vevo LAZR (FUJIFILM VisualSonics, Inc.) system. Figure S4e, Supporting Information shows representative in vivo ultrasound images of the orthotopic PDAC tumors in the absence and presence of the regular TPMIL + 690 nm (12.5 PDT dose product) treatment at day 70.

Our previous study on subcutaneous PDAC tumors using a chemotherapy‐free EGFR targeted PDT construct alluded to the fact that molecular targeted PDT modulates tumor collagen fractional area (Masson's Trichrome staining).^[^ ^14^ ^]^ A central point of focus of this study was the evaluation of the impact that combined molecular targeted PDT and irinotecan chemotherapy using the single TPMIL construct had on desmoplasia and survival in an orthotopic heterotypic PDAC model. Tumor collagen within desmoplastic PDAC tumors has been found to have a significant impact on tumor response, resistance, migration, invasion, and disease progression. Tumor collagens have been found to induce pro‐proliferative and anti‐apoptotic effects on PDAC cells, resistance to chemotherapy, promote adhesion, promote migration, and support PDAC cell viability.^[^ ^5^, ^44^, ^45^, ^46^, ^47^ ^]^

**Figure 5:**
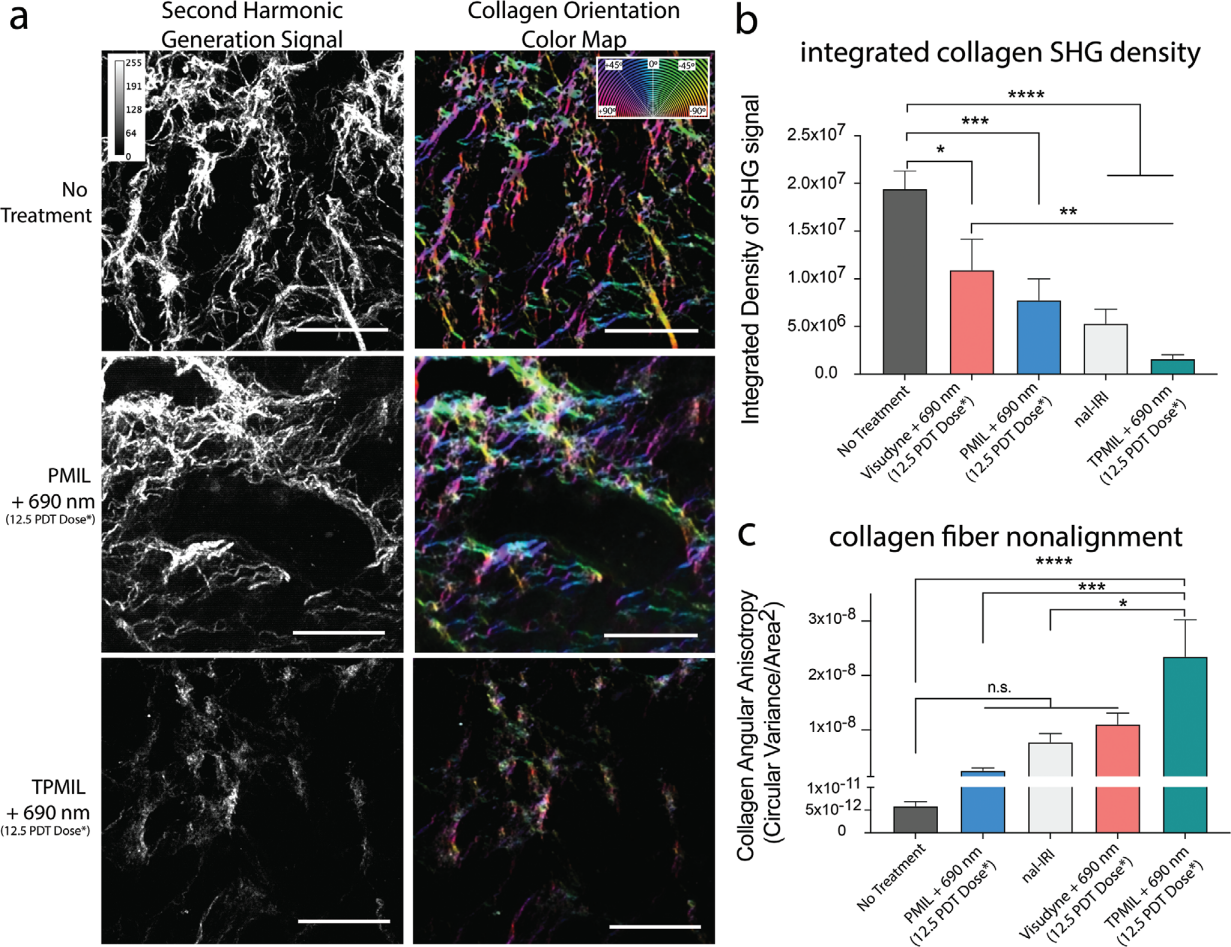
690 nm photoactivation of TPMIL remediates desmoplasia by disruption of collagen density and collagen alignment. a) Second harmonic generation images of control tumors and treated tumors 72 h following induction of treatment and respective collagen orientation color map images depicting the angles of collagen fibers within the image. Raw SHG signals are false colored white. b) 690 nm photoactivation of TPMIL significantly reduces collagen density (integrated density of SHG signal) and c) significantly increases collagen fiber angular anisotropy (collagen fiber circular variance/area^2^), as compared to all other treatment arms (scale bars are 100 mm; *PDT dose refers to the dose product of mg BPD eq. kg^−1^ × J cm^−2^; values are mean ± S.E.M.; statistical significance was calculated using one‐way ANOVA with a Tukey post‐test; *n* = 4–5 tumors; 40–50 tumor ROIs; *p ≤ 0.05, **p ≤ 0.005, ***p ≤ 0.001, ****p ≤ 0.0001).

SHG imaging is a non‐linear optical technique that has been widely used to characterize collagen density, structure, and alignment in pre‐clinical and patient tumors and fibrotic tissue, including PDAC patient tissue.^[^ ^25^, ^48^, ^49^, ^50^, ^51^, ^52^, ^53^ ^]^ In one study, SHG imaging revealed that collagen fibers in desmoplastic PDAC patient tumor tissue were longer and more aligned than collagen fibers in the tissue from healthy patients and patients with chronic pancreatitis.^[^ ^50^ ^]^ SHG was also used to characterize the stroma in a tissue microarray cohort from 165 patients.^[^ ^51^ ^]^ Of particular relevance to our study, another report assessing patient PDAC tissue with SHG imaging revealed that high collagen alignment in PDAC patients was in fact a negative prognostic indicator, whereby survival was significantly lower when tumor collagen was highly aligned.^[^ ^25^ ^]^ As such, we leveraged SHG microscopy to assess the collagen density and alignment within orthotopic PDAC tumor cryosections 72 h following treatment. SHG images were then analyzed using the OrientationJ plugin on ImageJ to quantify collagen density, generate collagen orientation color maps, and calculate collagen fibers angular anisotropy (nonalignment; Figure S6a, Supporting Information). **Figure** **5**a,b shows the raw false‐colored SHG signal in tumors treated with Visudyne + 690 nm (12.5 PDT dose product), PMIL + 690 nm (12.5 PDT dose product), nal‐IRI or TPMIL + 690 nm (12.5 PDT dose product), demonstrating a considerable and significant 91.8% decrease in collagen density in the TPMIL + 690 nm (12.5 PDT dose product) treated tumors, as compared to untreated controls. Figure 5a also shows the corresponding collagen orientation color maps that were further analyzed, revealing that collagen fiber nonalignment (circular variance in angular anisotropy) increased most significantly by 4.8 × 10^3^‐fold for the TPMIL + 690 nm (12.5 PDT dose product) treated tumors, as compared to the untreated control group (Figure 5c). SHG images and corresponding collagen orientation color maps, and tumors treated with Visudyne + 690 nm (12.5 PDT dose product) and nal‐IRI are presented in Figure S6b, Supporting Information. Interestingly, the mean collagen intensity per fiber unit area significantly decreased by ≈10% only in the TPMIL + 690 nm (12.5 PDT dose product) treated tumors (Figure S7, Supporting Information). This suggests that while other treatment arms also reduce the integrated collagen SHG density to a certain extent, only TPMIL + 690 nm (12.5 PDT dose product) treatment induces significant microstructural collagen perturbations. This hypothesis is supported by previous findings that demonstrate how physical modulation of collagen microstructure (e.g. enzymatic digestion) specifically reduces the SHG signal per fiber area.^[^ ^54^ ^]^

**Figure 6:**
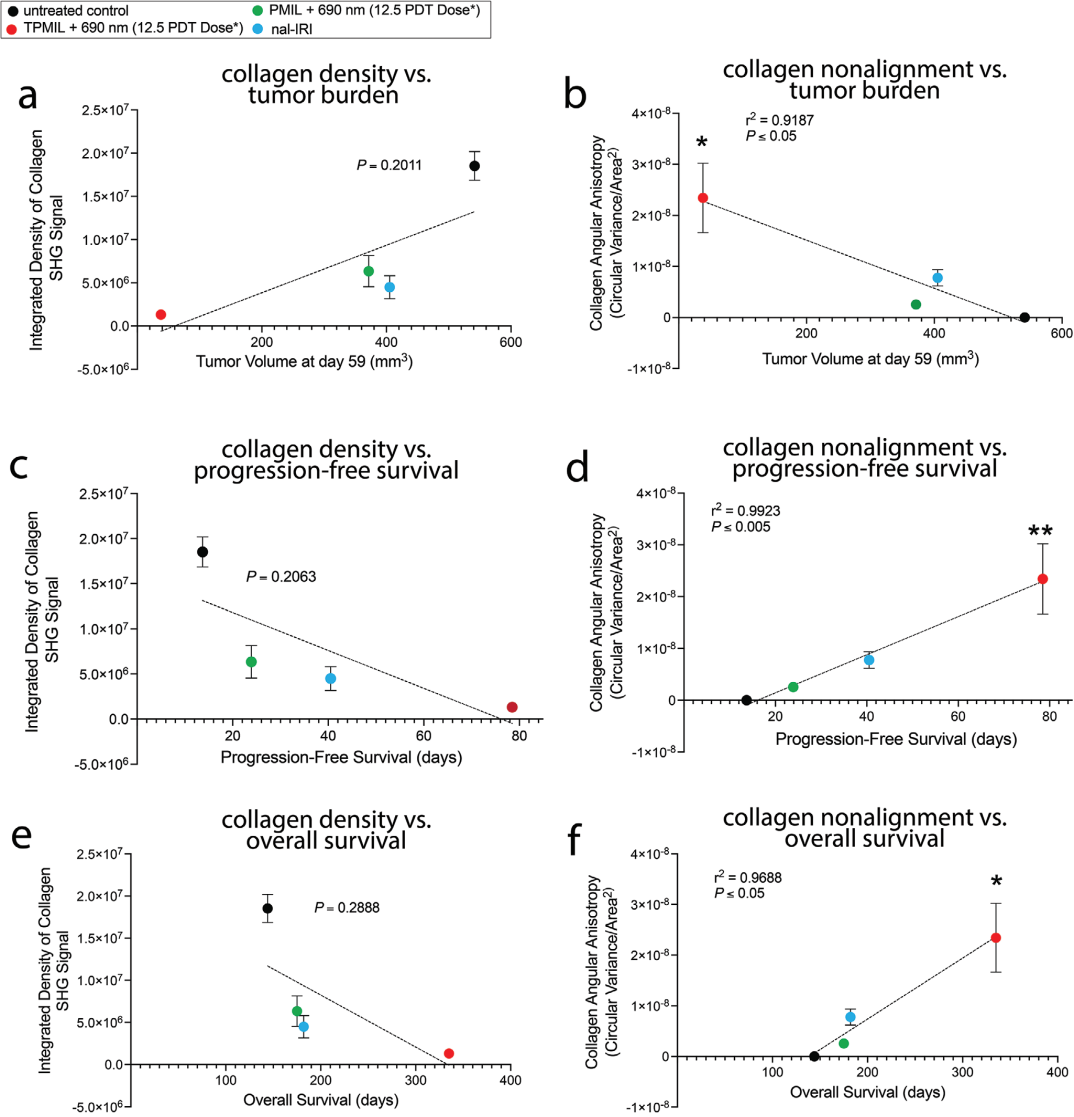
Remediation of desmoplasia correlates with reduction in tumor burden and prolonging of survival. Correlations between PDAC tumor burden at day 59 and a) collagen density or b) collagen nonalignment, between progression‐free survival and c) collagen density or d) collagen nonalignment, and between overall survival and e) collagen density or f) collagen nonalignment (*PDT dose refers to the dose product of mg BPD eq. kg^−1^ × J cm^−2^; values are mean ± S.E.M.; correlations were assessed using Pearson's *r* correlation coefficients; *n* = 4–5 mice per arm; 40–50 tumor ROIs per arm; *p ≤ 0.05, **p ≤ 0.005).

Remediation of desmoplasia by TPMIL + 690 nm (12.5 PDT dose product) was also associated with a reduction in tumor burden, progression‐free survival, and overall survival (**Figure** **6**). Although not statistically significant, the TPMIL + 690 nm (12.5 PDT dose product) treatment‐induced reduction in collagen density was associated with a reduction in tumor burden and an extension of progression‐free survival and overall survival. On the other hand, TPMIL + 690 nm (12.5 PDT dose product) treatment‐induced collagen fiber nonalignment negatively correlated with tumor burden (*r* ^2^ = 0.9187, *p* ≤ 0.05) and positively correlated with progression‐free survival (*r* ^2^ = 0.9923, *p* ≤ 0.005) and overall survival (*r* ^2^ = 0.9688, *p* ≤ 0.05). This suggests that concomitant molecular targeted photodamage, chemotherapeutic insult, and desmoplasia remediation upon TPMIL photoactivation provides the greatest benefit in controlling PDAC disease progression and provides the greatest survival benefit. These results suggest that TPMIL + 690 nm (12.5 PDT dose product) treatment‐induced collagen nonalignment is a significant positive prognostic indicator. This finding is consistent with the literature, which shows that high collagen fiber alignment specifically, rather than high collagen density, is a negative prognostic indicator in PDAC patients.^[^ ^25^, ^55^ ^]^

Here, remediation of desmoplasia by TPMIL + 690 nm (12.5 PDT dose product) is partially attributed to dose‐dependent photochemical degradation of collagen.^[^ ^18^ ^]^ At low PDT doses (<25 J cm^−2^), the stiffness of collagen hydrogels increases, likely due to collagen crosslinking. As the PDT dose increases beyond 25 J cm^−2^, the stiffness of collagen hydrogels decreases as the collagen is photochemically degraded. It is well established that singlet oxygen selectively oxidizes the collagen crosslink histidinohydroxylysinonorleucine and its precursor the amino acid histidine in ECM tissue.^[^ ^56^ ^]^ Oxidative destruction of histidine in collagen is also thought to influence the activity of collagen‐crosslinking lysyl oxidase, thereby disrupting the integrity of collagen matrices.^[^ ^56^ ^]^ Hydroxyl radicals, which are also generated by the BPD‐PC PS in the TPMIL constructs,^[^ ^37^ ^]^ are known to directly attack proline or 4‐hydroxyproline residues in collagen and fragment it to peptides.^[^ ^57^ ^]^ 4‐hydroxyproline in collagen is also a target for chemical attach by superoxide anion,^[^ ^58^ ^]^ a reactive molecular species also generated by the photoexcitation of BPD.^[^ ^59^ ^]^

In addition to the chemical degradation of collagen by TPMIL + 690 nm (12.5 PDT dose product), secondary factors also contributed to the reduction in collagen density and collagen fiber alignment. These secondary factors likely include the normalization of PCAFs and enzyme‐mediated stromal remodeling of tumor collagen in response to treatment.^[^ ^60^, ^61^ ^]^ PDT using BPD has been found to disrupt integrin–collagen adhesion and integrin signaling in fibroblasts, even without reducing integrin expression.^[^ ^62^ ^]^ This is most likely through structural perturbation of collagen by photochemical damage. This disruption of the integrin–collagen mediated adhesion and signaling is likely to contribute to the mechanistic therapeutic benefit of TPMIL + 690 nm (12.5 PDT dose product) treatment in this study. Interestingly, PDT using 5‐aminolevulinic acid has also been found to normalize and reverse the activation of CAFs, as demonstrated by a reduction in *α*‐SMA and FAP expression levels.^[^ ^63^ ^]^ This normalization of CAFs in turn reduces their production of collagen, and thus can also contribute to the remediation of desmoplasia observed in this study following TPMIL + 690 nm (12.5 PDT dose product) treatment. Furthermore, the activation status of PCAFs will also likely reduce the chemoresistance seen in PDAC cells that is typically attributed to activated CAFs.^[^ ^64^ ^]^ Sub‐lethal PDT on human vocal fold fibroblasts has also been shown to reduce their fibrotic effects.^[^ ^65^ ^]^ mRNA analysis on human vocal fold fibroblasts after sub‐lethal PDT demonstrated a significantly reduces transcription of genes related to the synthesis of ECM, namely collagen type I *α* 2 chain, collagen type III *α* 1 chain, fibronectin, and elastin. Sub‐lethal PDT also significantly increases transcription of genes related to matrix remodeling, namely matrix metallopeptidase 1.

Future studies will explore how the PCAF secretome is modulated and how the homeostasis of matrix remodeling enzymes (e.g. collagen‐crosslinking lysyl oxidase and collagen‐degrading matrix metalloproteases, etc.) is disrupted following TPMIL + 690 nm.^[^ ^60^, ^61^ ^]^ Furthermore, the collagen–integrin adhesion and signaling will be explored in both the PCAFs and PDAC cells following TPMIL + 690 nm. The activation status of PCAF following TPMIL + 690 nm will also be explored as a function of time and PDT dose. The interplay between stromal cells and immune cells will also be a critical point of focus in future studies to explore how photodynamic remediation of desmoplasia influences stromal‐induced immunosuppression in PDAC.^[^ ^61^ ^]^ This will be especially important for understanding how the desmoplasia remediation by TPMIL + 690 nm (12.5 PDT dose product) regulates the activity of tumor‐infiltrating T cells.^[^ ^66^ ^]^

## 4 Conclusions

Desmoplasia in PDAC contributes to treatment resistance, prevention of drug delivery, restriction of immune cell infiltration, pro‐tumorigenic signaling, tumor progression, and metastasis.^[^ ^2^, ^3^, ^4^ ^]^ Clinical studies have shown that stromal depletion alone is insufficient and in some instances detrimental to outcomes.^[^ ^9^, ^10^ ^]^ Nevertheless, desmoplasia is one of the predominant causes of PDAC resistance to even the most toxic chemotherapy regimen approved for PDAC patients, FOLFIRINOX, which fails to extend patient‐survival beyond 11.1 months.^[^ ^11^ ^]^ As such, transformative treatments such as PDT‐based combination therapies are in critical need for the effective management of PDAC in order to elicit multi‐modal assault on tumor tissue and modulate the tumor microenvironment without imparting overlapping toxicities.

In this study, we show for the first time that a single, EGFR‐TPMIL construct encapsulating a lipidated BPD molecule and irinotecan chemotherapy, is capable of simultaneously controlling tumor growth, remediating desmoplasia, and doubling survival. It does so through spatiotemporally‐synchronized chemotherapeutic and photodynamic insult to PDAC tissue, as well as reducing collagen density and increasing collagen fiber nonalignment. As is consistent with correlations in PDAC patient tumor tissue, increasing collagen fiber nonalignment in our study by TPMIL photoactivation significantly correlated with a lower tumor burden, prolonged progression‐free survival, and prolonged overall survival. Without EGFR molecular targeting, no anti‐tumor effect was observed and desmoplasia remediation was significantly less pronounced. This remarkable doubling of overall survival was achieved at a considerably low dose of irinotecan chemotherapy, which was only 8.1% of the total human dose of irinotecan given to patients—a dose that causes significant toxicities in up to 43% of patients.^[^ ^67^ ^]^ This unprecedented potentiation of chemotherapy is attributed to the co‐encapsulation process, which allows for simultaneous photodynamic and chemotherapeutic tumor tissue insult, as well as the molecular targeting which drives tumor cell endocytosis of the TPMIL constructs.

Being versatile in nature, the TPMIL constructs can be modulated to incorporate various therapeutics, thereby further supplementing the anti‐desmoplastic effects with multiple secondary or tertiary modalities. Of particular importance for future work, the co‐delivery of immunotherapeutic agents within the TPMIL constructs holds significant potential for more effective PDT‐induced control of metastatic PDAC. Given that desmoplasia prevents immune cell infiltration and contributes to an immunosuppressive tumor microenvironment, the TPMIL PDT approach can be used to simultaneously alleviate desmoplasia, promote immune cell infiltration, induce photodynamic immunogenic cancer cell death, re‐polarize tumor‐associated anti‐inflammatory M1 macrophages to pro‐inflammatory M2 macrophages, and co‐deliver immune‐potentiating immunotherapeutic agents. Our recent work also shows that triple‐receptor targeted nano formulations of lipidated BPD can potentiate T‐cell mediated attack of residual tumor cells following PDT.^[^ ^68^ ^]^ Introducing this approach to the TPMILs we present here can further strengthen their potential for complete eradication of heterogenous desmoplastic PDAC tumors that exhibit both intratumoral and intertumoral heterogeneity in receptor expression between patients, and augment the infiltration and responsiveness to T cell‐mediated tumor attack.

Multimodal nanotechnology‐assisted modalities like photoactivated TPMILs, that capitalize on the unparalleled biological effects of PDT, hold significant potential in transforming the clinical management of PDAC. By not limiting treatment to stromal modulation alone, this approach enables a simultaneous multi‐pronged attack on the physical, biological, and cellular components of desmoplastic PDAC, thereby minimizing the chances of treatment failure and augmenting the efficacy of the highly toxic chemotherapeutics currently used at considerably lower doses. Not only can this offer a significant advance over current and emerging PDAC treatments, but also holds significant potential for other cancer indications where desmoplasia is also problematic. Ultimately, a treatment modality that is safer, less toxic, more confined to tumor tissue, and more effective at both cancer tissue and stromal destruction, and which also extends PDAC patient survival, is of paramount importance and in urgent need.

## Conflict of Interest

The authors declare no conflict of interest.

## Supporting information

Supporting Information

## Data Availability

The data that support the findings of this study are available from the corresponding author upon reasonable request.
