# Supplementary material for: Remediating Desmoplasia with EGFR‐Targeted Photoactivable Multi‐Inhibitor Liposomes Doubles Overall Survival in Pancreatic Cancer
Source: Adv Sci (Weinh). 2022 Jun 24;9(24):2104594. doi: 10.1002/advs.202104594 (PMC9404396; doi:10.1002/advs.202104594)
Supplement: Supplementary file 1 — Supporting Information [file ADVS-9-2104594-s001.pdf]

Supporting Information

Remediating Desmoplasia with EGFR-Targeted Photoactivable Multi-Inhibitor Liposomes Doubles Overall Survival in Pancreatic Cancer

*Girgis Obaid, Shazia Bano, Hanna Thomsen, Susan Callaghan, Nimit Shah, Joseph W. R. Swain, Wendong Jin, Xiadong Ding, Colin G. Cameron, Sherri A. McFarland, Juwell Wu, Mark Vangel, Svetla Stoilova-McPhie, Jie Zhao, Mari Mino-Kenudson, Charles Lin and Tayyaba Hasan**

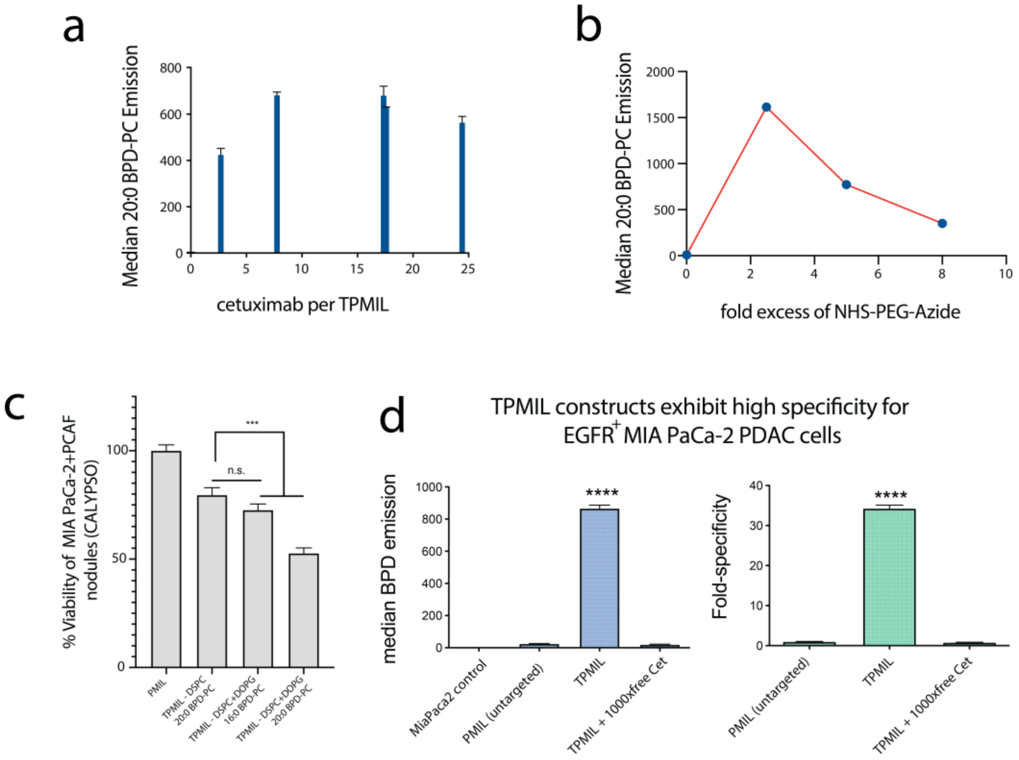

Figure S1. Flow cytometry data revealing the degree of MIA PaCa-2 cell binding of TPMIL construct varying the cetuximab density (a) and the fold-excess NHS-PEG-azide used to derivatize cetuximab for TPMIL constructs reacted at 100x cetuximab per construct (b). c) Viability of MIA PaCa-2 + PCAF nodules 72h following irradiation at 690 nm light at 40 $J.cm^{-2}$ and 150 $mW.cm^{-2}$ assessed using the live-dead technique and CALYPSO post-analysis. Results reveal that TPMIL construct containing 20:0 BPD-PC and post-inserted with DOPG are the most effective. d) TPMIL constructs exhibit high MIA PaCa-2 PDAC cell binding specificity, which can be competitively inhibited by the presence of an excess of free Cet. (Values are mean ± S.E.M.; statistical significance was calculated using One-Way ANOVA with a Tukey post-test; n > 3; *** = $P \leq 0.005$, **** = $P \leq 0.001$).

Table S1. Summary of the physical and chemical properties of TPMIL and untargeted PMIL constructs

Nanoconstruct	Hydrodynamic Diameter (nm)[(a)]	Polydispersity Index (P.D.I.) [(a)]	BPD : Irinotecan mass ratio[(a)]	Irinotecan entrapment efficiency (%)[(a)]	Cetuximab conjugation efficiency (%)[(a)]
TPMIL	143.4 ± 7.7	0.06 ± 0.02	1 : 37.9 ± 4.8	92.4 ± 17.1	30.1 ± 3.1
PMIL	144.8 ± 13.0	0.06 ± 0.03	1 : 38.2 ± 6.8	97.2 ± 3.7	*na*

[(a)]All values are presented as mean (± S.D.).

Table S2. *In vitro* therapeutic efficacy of all treatment arms assessed in this study.

Treatment Arm	IC_{50} (nM)[(a)]
TPMIL	237.07 ± 75.64
TPMIL + 20 $J.cm^{-2}$ 690 nm	3.53 ± 0.92
PMIL	1167.67 ± 256.54
PMIL + 20 $J.cm^{-2}$ 690 nm	74.29 ± 25.80
Visudyne + 20 $J.cm^{-2}$ 690 nm	1.91 ± 0.37
nal-IRI	36.11 ± 26.91 [(b)]

[(a)]All values are presented as mean (± S.D.). [(b)] values $\times 10^4$

Table S3. Summary of survival statistics for mice treated with TPMIL + 690 nm and other control groups

Treatment Arm	Progression-free survival (days; tumor volume ≤100 mm^3)	Median Survival (days)	Overall Survival (days)
Untreated Control	13.7	107.0	144.0
nal-IRI (20 $mg.kg^{-1}$)	40.5	171.0	195.0
Visudyne + 690 nm + nal-IRI (5 $mg.kg^{-1}$)	43.1	144.5	169.0
Visudyne + 690 nm + nal-IRI (20 $mg.kg^{-1}$)	37.5	146.0	204.0
PMIL + 690 nm	23.9	114.0	175.0
TPMIL + 690 nm	78.5	195.0	335.0

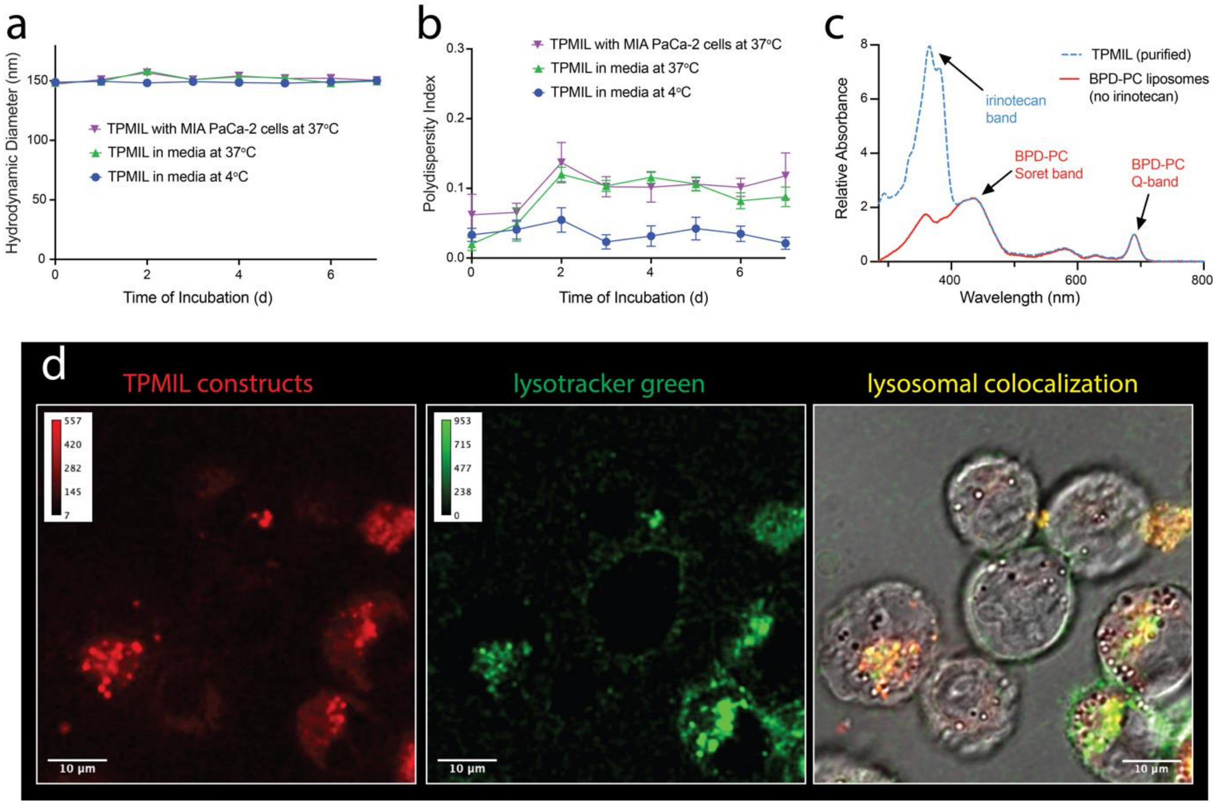

Figure S2. (a-b) TPMIL constructs remain stable for up to 7 days incubated in biological serum-containing media in the absence and presence of MIA PaCa-2 cells at 37°C. TPMIL constructs also remain stable in biological serum-containing media at 4°C. c) Relative absorption spectra in DMSO of BPD-PC liposomes without irinotecan and TPMIL constructs following active loading of irinotecan and purification of the constructs. The absorption spectrum of TPMIL constructs exhibits the characteristic absorption band of irinotecan and the characteristic Soret and Q-band of BPD-PC. d) confocal microscopy images of TPMIL constructs incubated with MIA PaCa-2 cells. Colocalization with lysotracker green suggests that the TPMIL constructs are internalized through receptor mediated endocytosis and are sequestered in endolysosomal compartments. (Values are mean ± S.E.M.; statistical significance was calculated using One-Way ANOVA with a Tukey post-test; scale bars are 100 nm; n = 3; * = P ≤ 0.05).

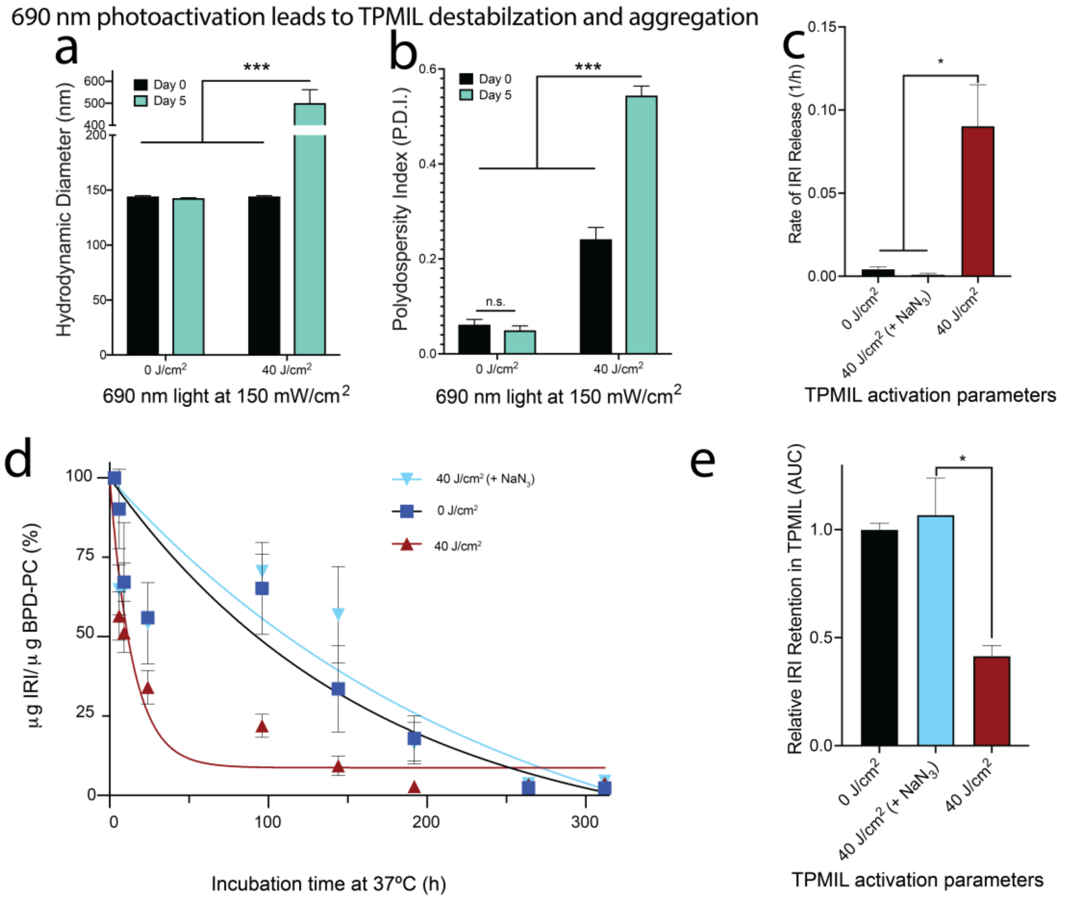

Figure S3. (a-b) TPMIL constructs destabilize and aggregate immediately following photodynamic activation as demonstrated by DLS analysis. c) Passive and phototriggered release of irinotecan from the TPMIL constructs at 37°C in 10% fetal bovine serum solutions in PBS. 690 nm activation of the TPMIL constructs (40 J.cm^{-2} at 100 mW.cm^{-2}) expedites the rate of irinotecan release (d) and decreases the relative retention of irinotecan (e). The presence of 100 mM sodium azide (NaN_3) completely inhibited phototriggered release of irinotecan. (Values are mean ± S.E.M.; statistical significance was calculated using One-Way ANOVA with a Tukey post-test; scale bars are 100 nm; n > 3; * = P ≤ 0.05, *** = P ≤ 0.005).

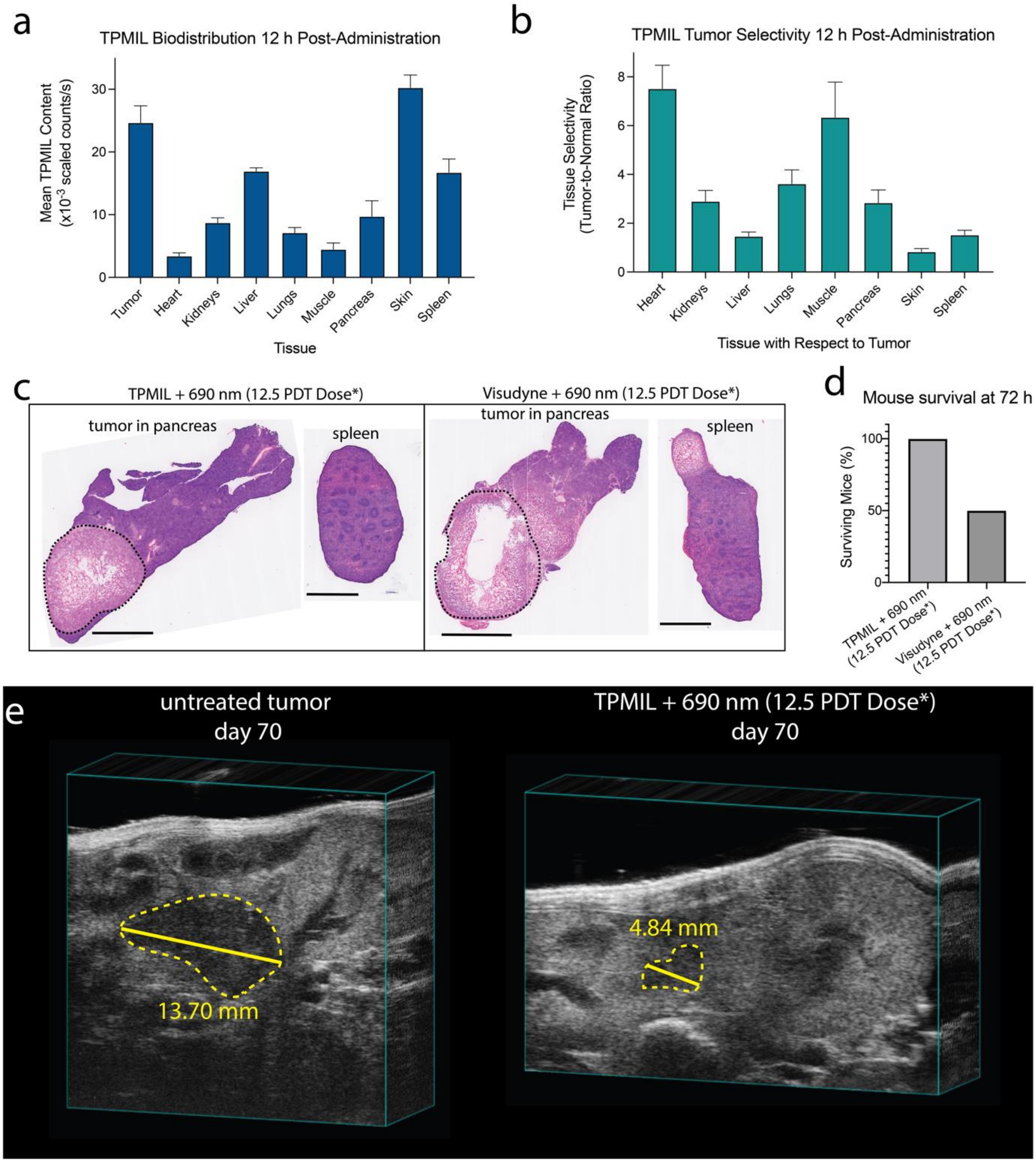

Figure S4. a) TPMIL construct full body biodistribution and b) tumor tissue selectivity at 12 h following administration. c) H&E tissue stains of PDAC tumors embedded within the pancreas and the nearby spleen 72 h following treatment with high dose (0.75 mg.kg^{-1} BPD equivalent, 150 J.cm^{-2}) TPMIL + 690 nm or Visudyne + 690 nm. The dashed black line depicts the tumor boundary within the pancreas revealing healthy pancreas and spleen necrosis only with the Visudyne + 690 nm treatment. (Scale bars are 2.5 mm). d) Mouse survival at 72 h following high dose (0.75 mg.kg^{-1} BPD equivalent, 150 J.cm^{-2}) TPMIL + 690 nm or Visudyne + 690 nm. e) representative 3D ultrasound images of orthotopic PDAC

tumors at d 70 in the untreated control arm (left) and TPMIL + 690 nm treated arm (right). (*PDT Dose refers to the
t of mg BPD eq.kg^{-1} x J.cm^{-2}; values are mean ± S.E.M, n=4.)

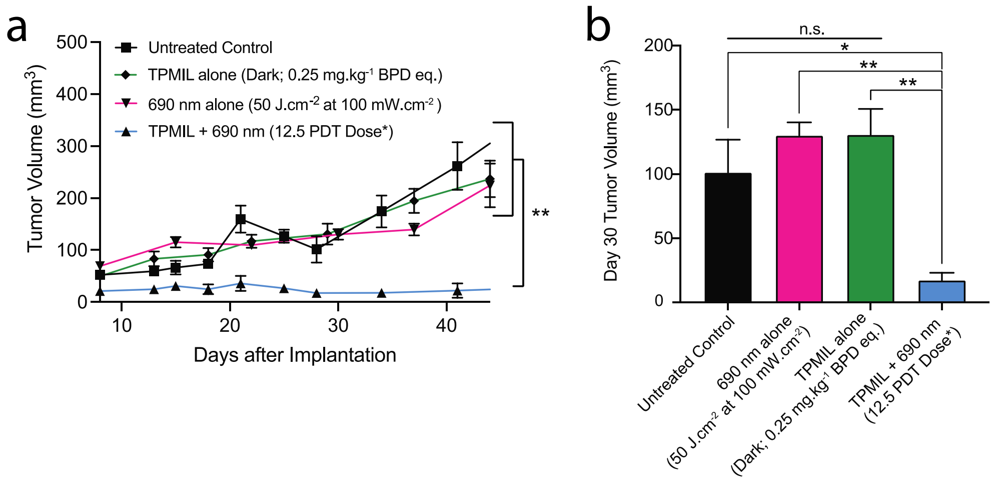

Figure S5. a) Photoactivation in the absence of TPMIL construct or TPMIL construct (0.25 mg.kg^{-1} BPD eq; 9.6 mg.kg^{-1} IRI equivalent) without photoactivation had no inhibitory effect on tumor growth, whereas the TPMIL construct + 690 nm (12.5 PDT Dose Product) effectively controlled tumor growth. b) Tumor volumes at day 30 following induction of treatment reveal that only TPMIL construct + 690 nm (12.5 PDT Dose Product) was capable of reducing the tumor burden. (*PDT Dose refers to the dose product of mg BPD eq.kg^{-1} x J.cm^{-2}; values are mean ± S.E.M.; statistical significance was calculated using One-Way ANOVA with a Tukey post-test; n = 4-8; * = $P \leq 0.05$, ** = $P \leq 0.005$).

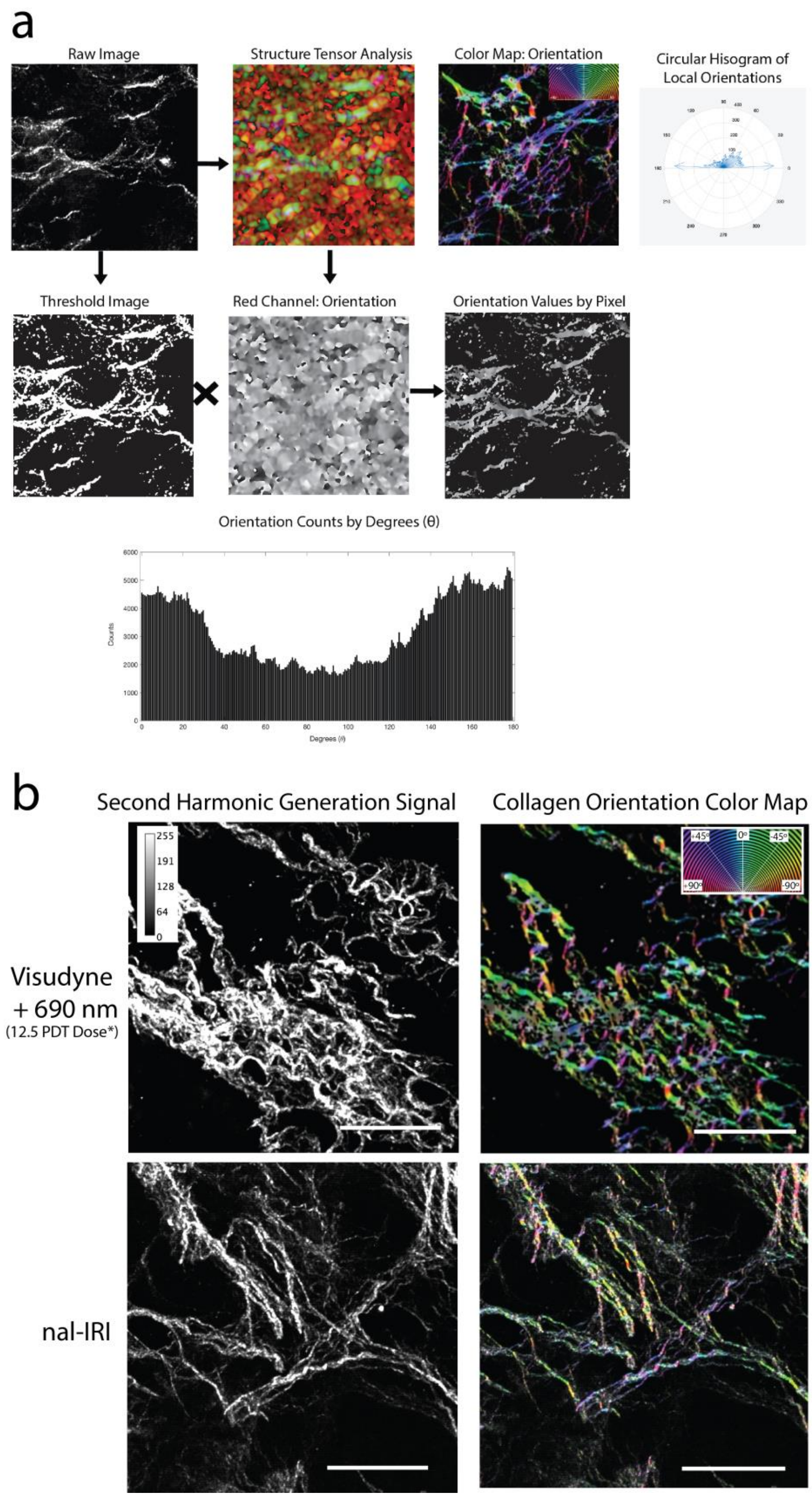

Figure S6. a) Second harmonic generation image analysis workflow for measuring collagen fiber orientation in tumor fiber bundles using the ImageJ plugin OrientationJ (http://bigwww.epfl.ch/demo/orientation/). b) Second harmonic generation images of Visudyne + 690 nm treated tumors and nal-IRI treated tumors 72 h following induction of

treatment (left). Respective collagen orientation color map images depicting the angles of collagen fibers within the SHG Images (right). Raw SHG signals are false colored white. (*PDT Dose refers to the dose product of mg BPD eq.kg^{-1} x J.cm^{-2})

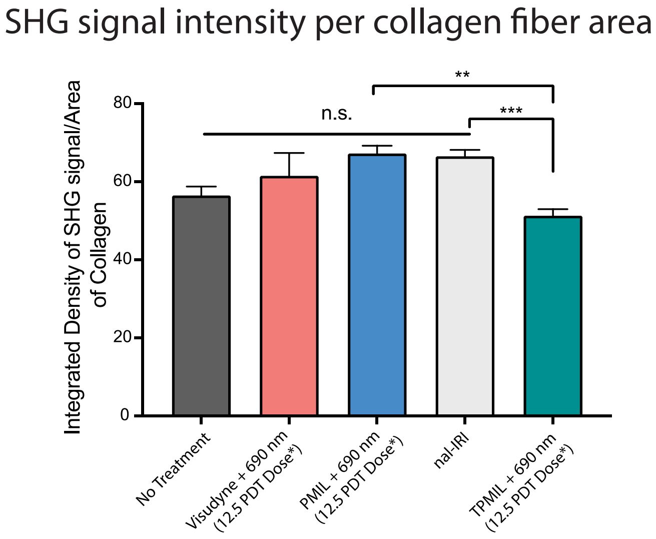

Figure S7. Analysis of second harmonic generation (SHG) images of tumor cross sections reveals that 690 nm photoactivation of TPMIL significantly reduces mean SHG signal intensity per unit area of collagen fibers (integrated density of SHG signal/area). (*PDT Dose refers to the dose product of mg BPD eq.kg^{-1} x J.cm^{-2}; values are mean $\pm$ S.E.M.; statistical significance was calculated using One-Way ANOVA with a Tukey post-test; n= 4-5 tumors; 40-50 tumor ROIs; * = $P \leq 0.05$, ** = $P \leq 0.005$, *** = $P \leq 0.001$, **** = $P \leq 0.0001$)
